# How the pH Controls Photoprotection in the Light-Harvesting Complex of Mosses

**DOI:** 10.1021/jacs.3c00377

**Published:** 2023-03-24

**Authors:** Laura Pedraza-González, Edoardo Cignoni, Jacopo D’Ascenzi, Lorenzo Cupellini, Benedetta Mennucci

**Affiliations:** Dipartimento di Chimica e Chimica Industriale, Università di Pisa, via G. Moruzzi 13, 56124 Pisa, Italy

## Abstract

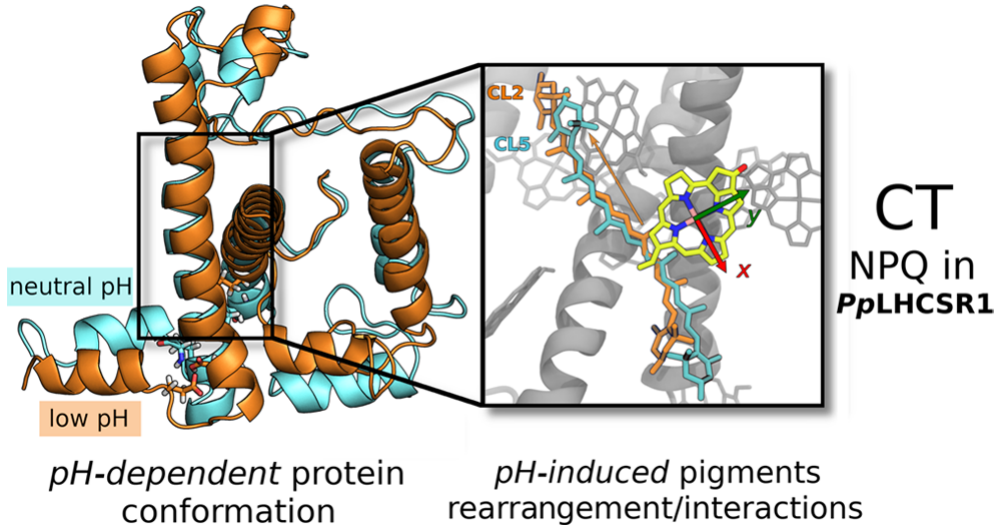

In response to varying light conditions, light-harvesting complexes (LHCs) switch from a light-harvesting state to a quenched state to protect the photosynthetic organism from excessive light irradiation in a strategy known as nonphotochemical quenching (NPQ). NPQ is activated by an acidification of the thylakoid lumen, which is sensed directly or indirectly by the LHC, resulting in a conformational change of the complex that leads to the quenched state. The conformational changes responsible for NPQ activation and their connection to specific quenching mechanisms are still unknown. Here, we investigate the pH-triggered conformational changes in the light-harvesting complex stress-related (LHCSR) of mosses. By combining constant-pH molecular dynamics and enhanced sampling techniques, we find that the pH sensitivity of the complex is driven by the coupled protonation of three residues modulating the conformation of the short amphipathic helix placed at the lumen side of the embedding membrane. Combining these results with quantum mechanics/molecular mechanics calculations, we show that the quenching mechanism sensitive to the pH goes through a charge-transfer between a carotenoid and an excited chlorophyll, which is controlled by the protein conformation.

## 1 Introduction

In plants, mosses, and green algae, sunlight is collected by light-harvesting pigment–protein complexes (LHCs) and rapidly funneled to the reaction centers (RCs) of photosystems. This is made possible by the aggregate of interacting pigments contained in LHCs, chlorophylls (Chls), and in minor part carotenoids (Cars), which create a path for the absorbed energy to efficiently move in space and reach the RC via excitation energy transfer (EET) processes. This functioning, however, is adaptable to the changing light conditions.^1^ In fact, under excess light conditions, the LH process is replaced by quenching of the excited chlorophylls and the dissipation of the excess energy into heat in a process called nonphotochemical quenching (NPQ).^1−4^ The major and most rapid NPQ component is energy-dependent quenching (qE), which is triggered by a pH drop in the thylakoid lumen.^2−4^ In fact, increasing light intensity causes lumen acidification and thus the generation of a pH gradient across the thylakoid membrane, which acts as a reporter of the amount of absorbed energy.

Many details of how qE works at a molecular level are still missing, but it is now clear that plants behave differently from green algae, and these differences can be related to the specific place of each organism in the evolutionary chain.^4,5^ In green algae, the lumenal pH drop is sensed directly by specific LHCs, namely, light-harvesting complex stress-related (LHCSR) proteins, which also quench excitation.^6−11^ Conversely, in plants, a pigmentless protein, the Photosystem II subunit S (PsbS), is needed to sense lumenal pH.^2−5^ In mosses, which are intermediate in the evolution between green algae and vascular plants, both PsbS-mediated activation and direct activation seem to be possible.^7,12,13^ Interestingly, there are analogies between PsbS and LHCSR, specifically regarding pH-sensing lumenal residues.^14^ These amino acids are suggested as probes of the variations of lumenal pH, which under physiological conditions ranges from ∼4.5 to ∼7.5 under high and weak light exposure, respectively. Whatever the pH sensing mechanism, LHCs are able to convert between a LH and a quenching state by changing their conformation.^15,16^ In plant LHCs, the conformational change responsible for the switching is triggered by interaction with PsbS,^17^ whereas in LHCSR proteins, the conformational switch is directly activated by the protonation of lumen-exposed residues.^11,18,19^ Indeed, it is generally accepted that a different protonation state of a few titratable sites of a protein may contribute to a change in its conformation and function.^14^

But how does the conformational change activate quenching? A credited hypothesis is that the rearrangement of the protein scaffold leads to a different interaction between the embedded pigments, as they change their disposition.^20−22^ The changed interaction can then activate or deactivate charge-transfer (CT) and/or excitation energy transfer (EET) processes, which finally lead to the quenching.^22^

The CT mechanism consists of an electron transfer from a carotenoid to an excited chlorophyll in its Q_*y*_ state, followed by charge recombination to the global ground state^23−30^ In the EET mechanism, instead, the excitation energy of the excited Chl is transferred nonradiatively to the low-lying excited state (S_1_) of a nearby Car,^3,22,31−36^ which finally decays to the ground state dissipating the energy into heat. In either case, the quenching mechanism should be easily switchable by a change in the protein conformation. Otherwise, it would not be possible to observe drastic variations in excited-state lifetimes of LHCs upon changing conditions.

In order to gain further understanding of the NPQ process, focusing on mosses could be a valid choice since they express both LHCSR and PsbS, whereas plants lack the former and algae lack the latter. Moreover, since most of the NPQ activity in mosses is performed by LHCSR^7−11,14,37−39^ this complex is the most promising one for studying NPQ. As LHCSR shares both characteristics of other LHCs and PsbS, gaining insight into the NPQ triggering in LHCSR could also help understand how NPQ is regulated in the other LHCs.

**Figure 1:**
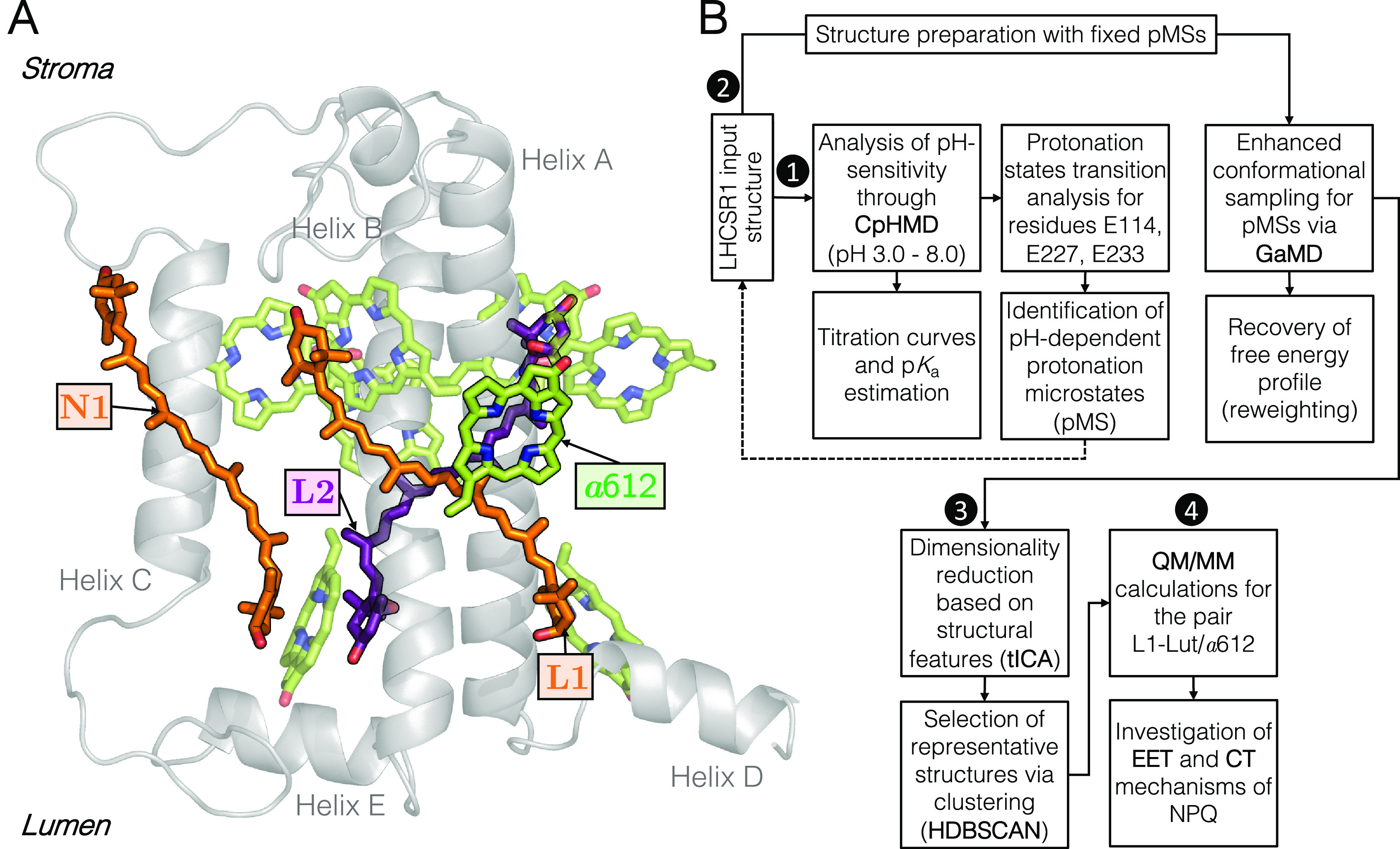
(A) General structure and pigment composition in *Pp*LHCSR1.^40^ The complex contains 8 chlorophylls *a* (green), two luteins located in the L1 and N1 (orange) sites, and a violaxanthin (purple) in the L2 site. Labels for all of the Chls are shown in Figure S11. (B) Multistep computational protocol used to investigate pH-induced conformational changes in *Pp*LHCSR1 and their link to NPQ. White numbers in black circles represent each step: (1) analysis of pH sensitivity of the lumen and identification of pH-induced protonation microstates (pMSs); (2) enhanced exploration of conformational space for selected pMSs; (3) characterization of pH-induced conformations of the protein at low- and high-pH conditions; (4) investigation of the NPQ mechanism.

Unfortunately, no experimental structural information is available on any protein in the LHCSR family. However, in our group, a homology modeling strategy coupled to molecular dynamics (MD) has allowed us to construct a three-dimensional atomistic model of LHCSR1 of the moss *Physcomitrella patens* (*Pp*LHCSR1).^40^ Such a model features a monomer of LHCSR1 equilibrated in a simulated native-mimicking membrane and consisting of three transmembrane α-helices (A, B, and C) and two amphipathic helices at the lumenal side (D and E), along with unfolded loops connecting them, for a total of 163 amino acid residues. As depicted in Figure 1A, LHCSR1 binds eight chlorophylls *a* and 3 carotenoids: lutein in sites L1 and N1 and violaxanthin in site L2.

Starting from that initial structure of LHCSR1, in this paper, we explored the conformational space of the complex, characterized the conformations mostly populated at neutral and low pH, respectively, and connected the geometrical specificities of the different conformations with the EET and CT mechanisms of quenching. As illustrated in Figure 1B, this analysis is the result of an integrated approach that combines constant-pH Molecular Dynamics (CpHMD),^41^ Gaussian Accelerated Molecular Dynamics (GaMD),^42,43^ dimensionality reduction, and clustering with quantum mechanics/molecular mechanics (QM/MM) calculations of the two quenching processes.

Through this approach, we identified which lumenal residues are likely to change protonation state under different pH conditions and characterized the corresponding conformations. Our simulations showed that lowering the pH down to 5 results in the population of different conformations with respect to pH 7.5, as previously seen in single molecule experiments.^18,19^ Moreover, we found that these different conformations can indeed distinguish between quenched and unquenched states of the complex through the activation of a charge-transfer mechanism in the Lut-Chl pair of the L1 site.

## 2 Results and Discussion

### 2.1 Identification of the Ionizable Residues Involved in Lumenal pH-Sensing

**Figure 2:**
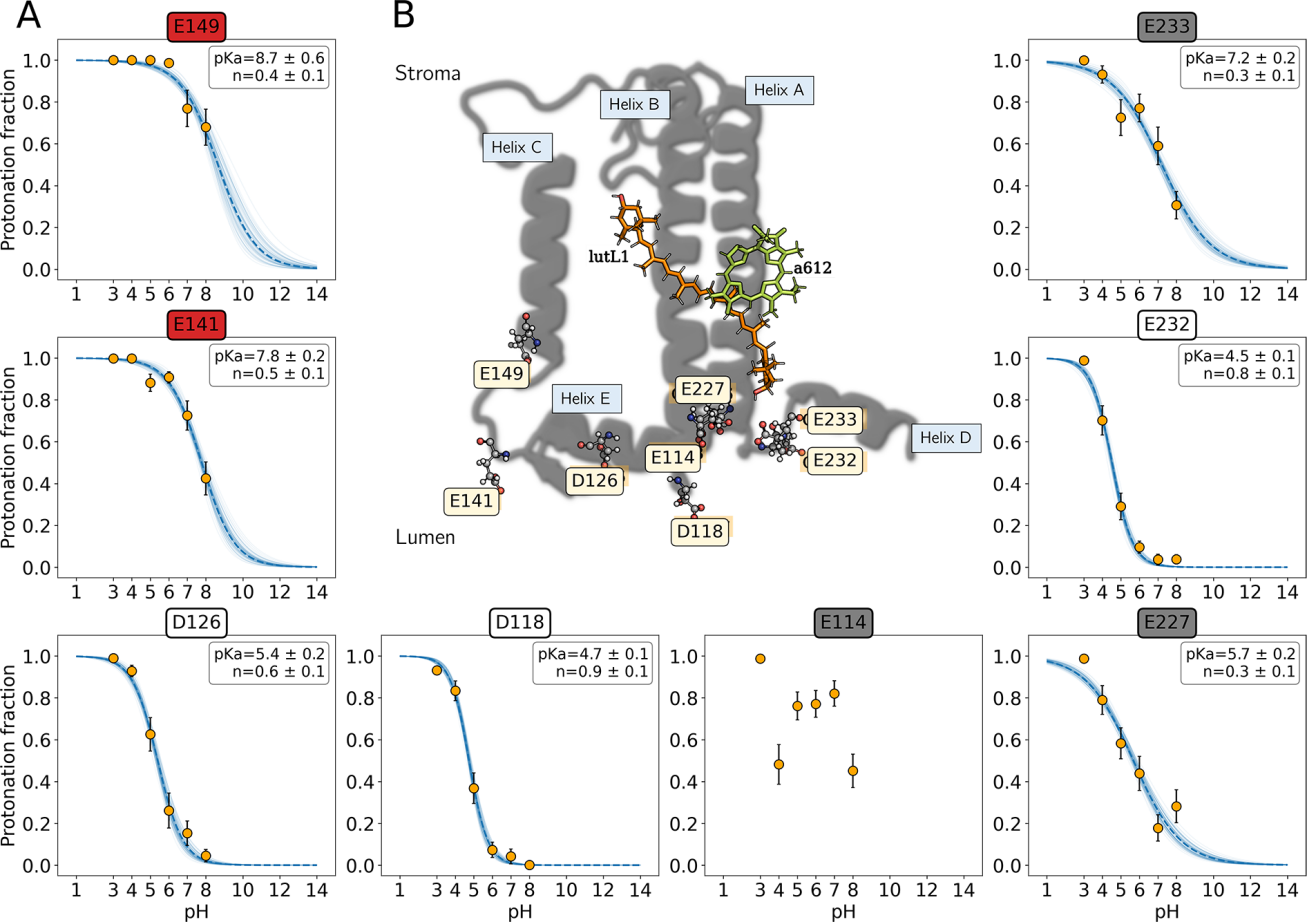
pH sensivity in the thylakoid lumen of LHCSR1. (A) Simulated titration curves of lumen-exposed ionizable acidic residues. Each point is computed as the average of two independent replicas of 600 ns each. The blue dashed curve represents the fit of the CpHMD data to the Hill equation to obtain the calculated p*K*_a_ values and Hill coefficients (*n*).^44−50^ The errors associated with the points, the p*K*_a_ values, and the Hill coefficients are two times the standard errors as estimated from 1000 bootstrap samples. Blocking of the trajectory was used when computing the bootstrap standard errors so as to correct for correlation in the data. Residues labeled inside red, white, and gray boxes are considered as protonated, deprotonated, and coupled, respectively. (B) General structure of a LHCSR1 monomer. The protein structure is represented as gray cartoons, and putative protonatable sites considered in CpHMD simulations are represented as ball-and-stick.

In this section, we investigate the function of LHCSR1 as a sensor of lumen pH by means of explicit-solvent CpHMD^41^ simulations at 6 different pH values. A range from 3.0 to 8.0 has been explored with a spacing of one unit, running two independent replicas of 600 ns each for a total of 7.2 μs. In such simulations, we considered as target pH-sensing groups exclusively the eight ionizable acidic residues predicted by the model of Guarnetti Prandi et al.^40^ to face the thylakoid lumen, as illustrated in Figure 2. These are Glu 114 (helix B), Asp 118 (loop helix B–D), Asp 126 (helix E), Glu 141 (loop helix C–E), Glu 149 (helix C), Glu 227 (helix A), Glu 232 (loop helix A–D), and Glu 233 (helix D). Notice that in the following we will use the amino acids’ one-letter symbols.

#### 2.1.1 Titration Curves and p*K*_a_ Prediction

By computing the protonation frequency along CpHMD simulations for the eight target residues at each pH value, we reconstructed their titration curves and estimated their p*K*_a_ values using the Hill approximation^45,47,49^ (section S2). Such titration curves represent a first screening to identify the residues that can act as pH sensors in the lumen of LHCSR1. A similar analysis reported for PsbS^51^ revealed that the responsiveness to thylakoid lumen acidification can be explained by the unusually high p*K*_a_ of lumen-faced aspartate and glutamate residues with respect to their reference values in water, which are several pH units below the range of lumenal physiological pH.^51−53^ Therefore, we are mainly interested in those residues of LHCSR1 that exhibit a p*K*_a_ strongly shifted from their values in water (i.e., Glu = 4.3, Asp = 3.9),^54^ as residues with a small p*K*_a_ shift would not be able to respond to lumen acidification around pH 5.5–6.^10^ A first inspection of titration curves in Figure 2B reveals that this is the case for residues D118 (p*K*_a_ = 4.7) and E232 (p*K*_a_ = 4.5), which would have a low probability to be protonated at pH > 5 and, consequently, are not involved in pH sensing of LHCSR1.

By contrast, large shifts (more than one pH unit) in the calculated p*K*_a_ were observed for D126 (p*K*_a_ = 5.4), E141 (p*K*_a_ = 7.8), E149 (p*K*_a_ = 8.7), E227 (p*K*_a_ = 5.7), and E233 (p*K*_a_ = 7.2). These residues have a high probability to be protonated at pH > 5 and will be analyzed further. On the other hand, we deal with the impossibility of computing the p*K*_a_ for residue E114, which presents a nonmonotonic “up-and-down” titration curve (see Figure 2A). This behavior is characteristic of titratable groups that are coupled to one or more neighboring ionizable groups and do not tritrate independently.^51^

#### 2.1.2 Coupling between Lumenal Titrable Groups

**Figure 3:**
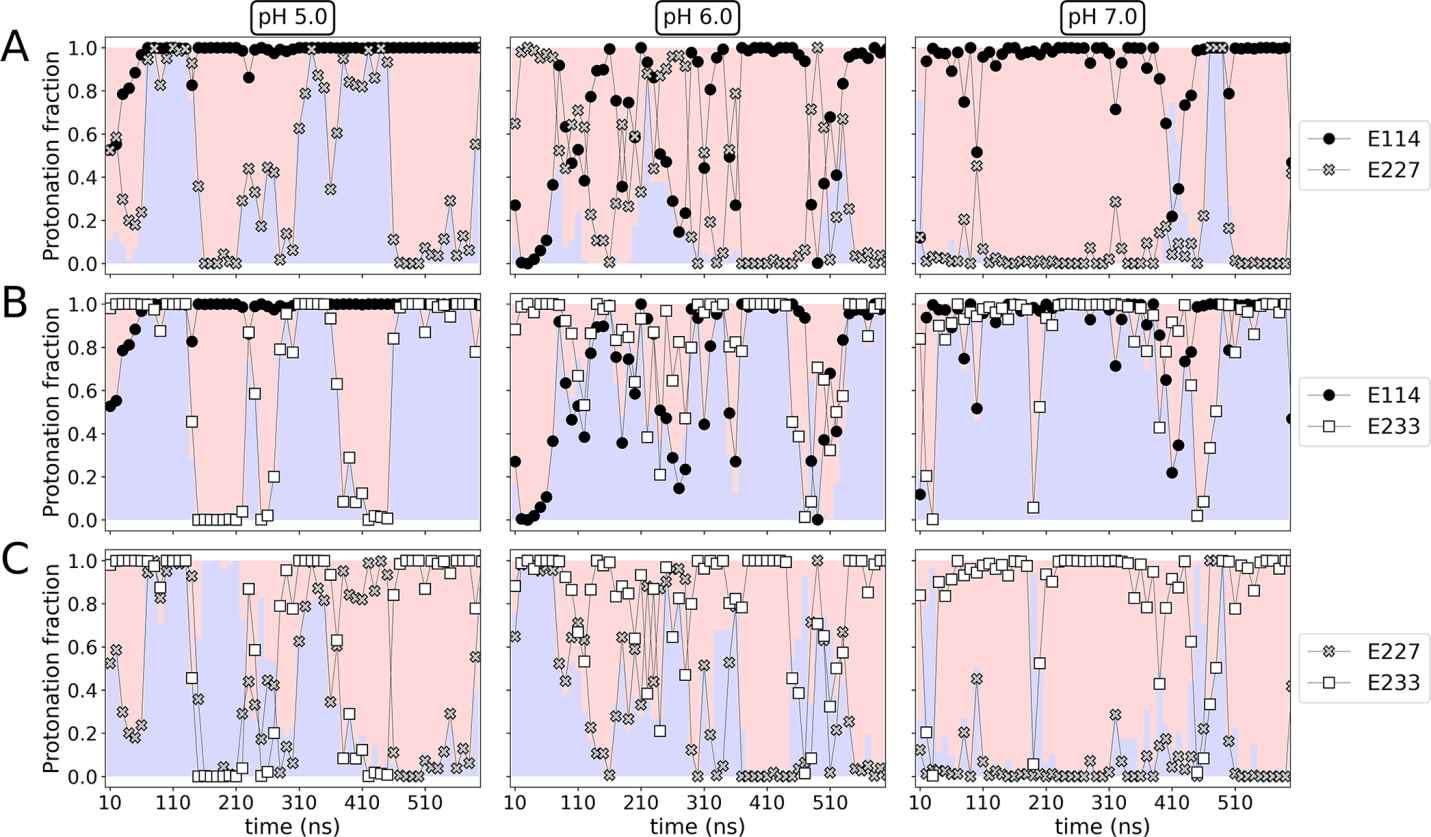
Protonation fraction for ionizable groups strongly coupled: (A) E114/E227, (B) E114/E233, (C) E227/E233. Blue and red shadows represent positive and negative correlations, respectively, computed as the average of 10 ns. Data correspond to one of the replicas.

We first evaluate the possible correlation (i.e., positive or negative) between the pairs E114/E227, E114/E233, and E227/E233. We note that a positive correlation implies the interaction between more than two residues, causing two of them to be simultaneously both neutral and both charged. Conversely, a negative correlation yields either a charged/neutral or neutral/charged joint state.^51^ To monitor how the ensemble of protonation states for each of the above-defined pairs evolves during the course of CpHMD simulations, in Figure 3 we plot their protonation fraction computed as averages over 10 ns windows at pH 5, 6, and 7.

As observed in Figure 3A, at pH 5 (left panel) the pair E114 (helix B)/E227 (helix A) presents either a positive or a negative correlation, and therefore, it is not possible to establish a specific pattern of coupling. At pH 6 (middle panel) and 7 (right panel), a strong negative correlation becomes evident; i.e., either E114 or E227 is deprotonated. At pH 6 the residues in the pair seem to constantly exchange their protonation states, at pH 7 E114 is protonated and E227 is deprotonated most of the time. These results suggest that at pH > 5 the residues in the pair tend to share the proton acceptor/donor role, remaining hydrogen bonded and acting as a “glue” between helices A and B.

For the pair E114 (helix B)/E233 (helix D), at pH 5 (Figure 3B, left panel), the situation is similar to what was discussed for E114/E227, i.e., either a positive or a negative correlation along the simulation. However, upon comparison of Figure 3A,B, it is possible to notice a pattern shared by the pairs E114/E227 and E114/E233: when one pair presents a positive correlation, the other one exhibits a negative correlation. This behavior provides insights into the coupling between the three analyzed residues. At higher pH (6 and 7), residues E114 and E227 have a different protonation state (i.e., when one is protonated the other is deprotonated) most of the time, resulting in a stronger coupling with respect to residues E114 and E233, which instead have the same protonation state. Lowering the pH instead increases the interaction between E114 and E233.

Analysis of the pair E114 (helix B)/E232 (loop B–D) reported in Figures S1 and S2 (see section S3) reveals that, irrespective of either the pH or the protonation state of E114, E232 tends to be always deprotonated, consistent with its predicted p*K*_a_ of 4.5 (Figure 2A). Moreover, the two aspartates D118 and D126 are mostly deprotonated (Figures S1 and S2), whereas the opposite is found for E141 and E149, which are mostly protonated regardless of pH, indicating that none of these residues are coupled to E114.

Summarizing, the above results complement our analysis of the titration curves and p*K*_a_ presented in section 2.1.1 and point out that D118, D126, E141, E149, and E232 most likely are not involved in the pH sensing of LHCSR1. Notably, mutagenesis analysis on *Chlamydomonas reinhardtii* LHCSR3 found that several lumenal acidic residues are not essential for pH sensing.^55^ Analogous experiments on *Pp*-LHCSR1 are needed to confirm our predictions. Most importantly, our analysis suggests a strong correlation within the triad E114, E227, and E233, modulated by pH. The interactions between those residues might be influenced by the pH in a nonobvious way via the formation of specific protonation patterns (i.e., protonation microstates, pMS). As a consequence, such pH-sensing could result in a pH-dependent conformational switch involving helices A, B, and D. The latter is of particular interest since experimental studies carried out on LHCSR proteins from other species^37,56^ indicate that helix D acts as a pH sensor in the lumen and can respond by tuning NPQ according to the acidity of the lumenal layer.

#### 2.1.3 pH-Dependent Protonation Microstates

**Figure 4:**
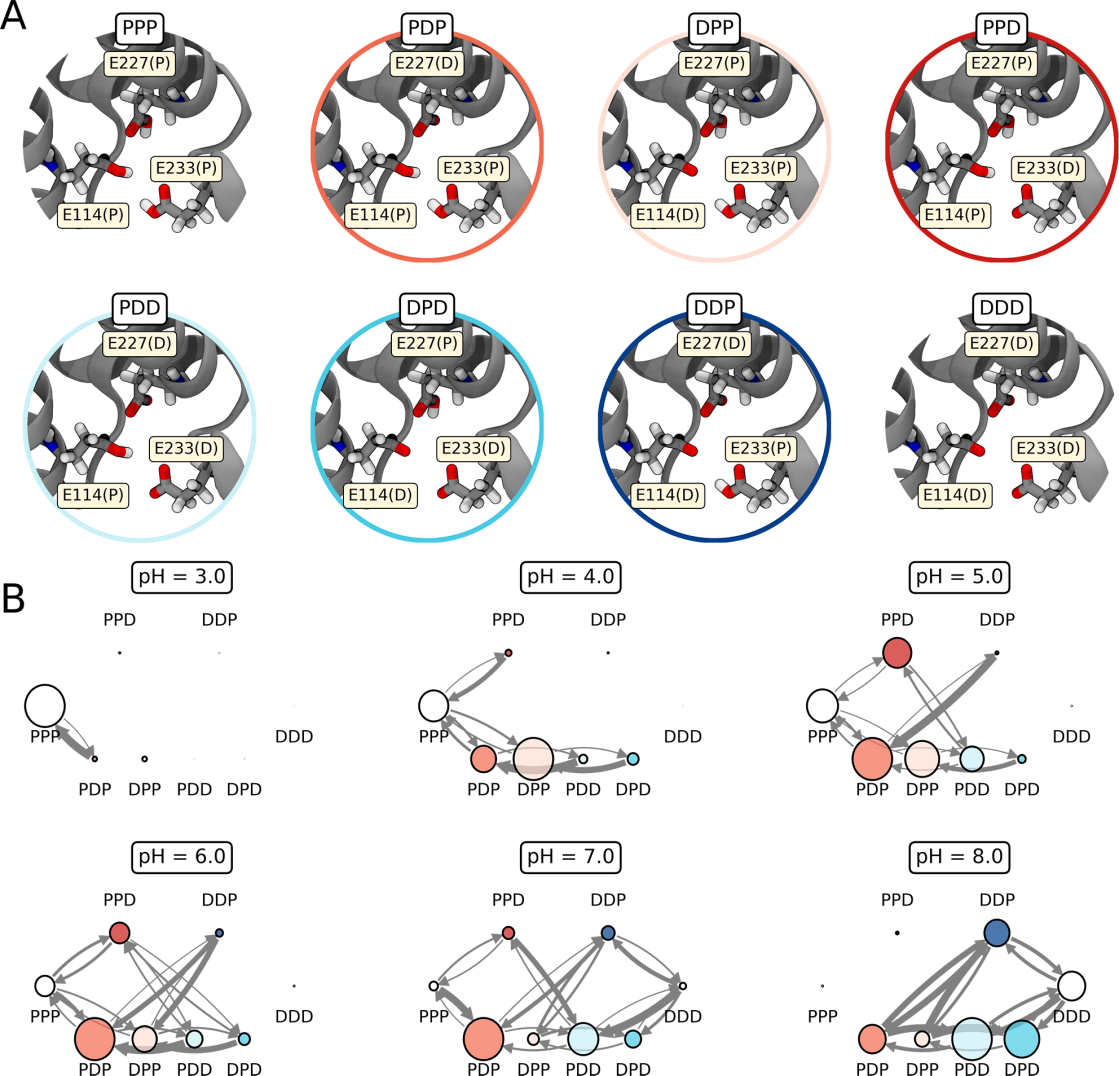
Protonation state transition analysis. (A) Definition of the eight pMSs formed by the triad E114/E227/E233, shown here on a single structure. Microstates that have been selected for further analysis (DDP, PDD, DPP, and PDP) are highlighted by a colored circle. (B) Analysis of protonation state population and transitions of the pMSs at each computed pH. Circle size denotes state population, and arrow thickness indicates transition probability from one protonation microstate to the other. Color-filled circles correspond to the pMSs selected for further analysis (see also panel A). The analysis considers two independent replicas of CpHMD simulations (0.6 μs each pH).

In order to identify the pMSs that are dominant at each pH, we have estimated a Markov state model based on the data obtained with the CpHMD.^57^ The Markov model provides the transition probabilities between all the pMSs (see Figure 4A) at a given pH, as well as the population of each pMS, represented as a network plot in Figure 4B. Further details on the construction of the Markov model are provided in section S4.

Figure 4B depicts the network plot for each evaluated pH. As observed, there is a distinct pH-dependent pattern that describes the population and transition changes of the pMSs when the pH is raised from 3.0 to 8.0. At extremely low pH (i.e., 3.0), the completely protonated (PPP) scenario dominates, whereas at high pH (i.e., 8.0) multiple pMSs involving two or three deprotonated residues are prevalent.

We focus on identifying the relevant pMSs in the pH range compatible either with stress conditions (pH ∼ 5.5) or with low-light mimicking conditions (pH ∼ 7.5).^11^ In our model, the former can be represented by transitions computed at pH 5–6, while the latter by transitions computed at pH 7–8.

As observed in Figure 4B, at stress conditions, the dominating pMSs are double-protonated pMSs, namely, PDP, DPP, PPD, and, to a minor extent, PDD (see Figure 4A). We remind the reader that such notation corresponds to either the protonated (P) or deprotonated (D) form of residues E114, E227, and E233, respectively.

We note that PDP remains considerably populated also at high pH, while the population of PPD largely reduces at pH ≥ 7 (see Figure 4B). As a matter of fact, the population of PPD is confined in a small pH range, namely, 5–6, which is the one compatible with quenching conditions (pH ∼ 5.5). Among the pMSs populated at stress conditions, PPD is thus the one showing the highest sensitivity to pH changes.

As expected, at low-light mimicking conditions (pH ≥ 7.0), the population of singly deprotonated pMSs increases, even if, as already observed, the population of the doubly deprotonated PDP remains relevant. The most populated pMSs are PDD and DPD, while DDP shows the highest degree of interconnections with PDP. As such, DDP behaves as a bridge between pMSs found in acidic and basic conditions.

This transition network analysis reveals that all singly or doubly protonated pMSs are relevant in the range of physiological pH. On the basis of this analysis, we have chosen to investigate these pMSs more in detail. Microstates such as PPP and DDD were excluded from the analysis: the former because it seems to be less relevant at pH ∼ 5.5 and the latter because it is only populated at high pH conditions (8.0).

### 2.2 pH-Dependent Conformational Changes in LHCSR1

We now analyze in detail whether different protonation patterns show different conformational preferences. To this end, we performed a new set of MDs using the pMSs established in the previous analysis (see the colored circles in Figure 4A,B); i.e., the protonation patterns are now fixed. According to the computed p*K*_a_’s (see Figure 2), in all cases E232, D118, and D126 are deprotonated, whereas E141 and E149 are protonated. To guarantee extensive conformational sampling, we employed an enhanced sampling technique, Gaussian Accelerated Molecular Dynamics (GaMD)^42,43^ (see Figure 1B). GaMD is an unconstrained enhanced sampling methodology, which applies a bias independently of selected collective variables. We run several independent 2 μs long GaMD replicas for each pMS for a total of 38 μs of enhanced dynamics. Details of the simulation protocol are provided in section 4.2.

In order to collectively analyze all GaMD simulations on equal footing and to make sense of the resulting conformational ensemble explored, we have employed time-lagged Independent Component Analysis (tICA)^58−60^ to transform selected intermolecular coordinates into a two-dimensional description of the conformations. Given the strong evidence suggesting that the switch between light-harvesting and quenched structures is located at the level of the C-terminal domain,^8−11,14,38,39,53,56^ we concentrated our analysis on that portion of the protein. Specifically, we have selected distances and side chain dihedrals involving the three residues characterizing the pMS (E114, E227, and E233) and distances and angles describing the relative positions of helices A, B, D, and E. More details are provided in section S6.

**Figure 5:**
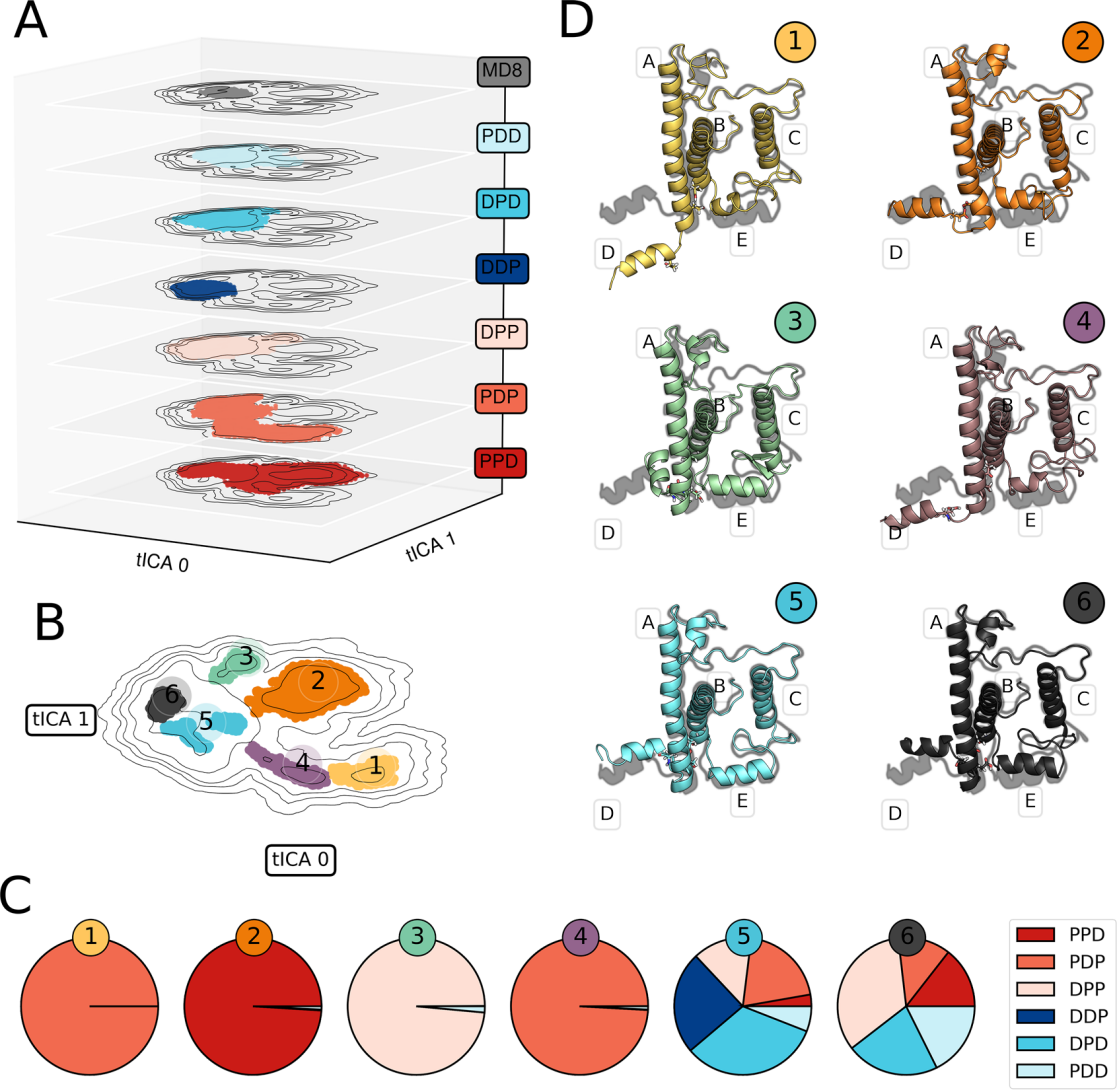
Captured conformational space of the pMSs. (A) Individual GaMD simulations for each pMS are projected onto the first two principal tICA components. The first 200 ns are excluded. (B) Clustering in tICA space. Each cluster is shown in a different color and indicated with its label. (C) pMSs composition of each cluster. (D) A representative structure of each cluster, selected as the one closest to the average one, and the unbiased template (MD8, gray shadow).

Figure 5A depicts the conformational landscape explored by each pMS, projected onto the first two tICA coordinates. We also plot the projection of the unbiased simulation of Guarnetti Prandi et al.^40^ featuring the DDD pMS, hereinafter referred to as MD8 to be consistent with ref (40).

As shown in Figure 5A, each pMS explores a portion of the conformational space with some overlapping areas where more than one pMS is present. Strikingly, singly protonated PDD, DPD, and DDP explore a region similar to the one sampled by the reference MD8, while doubly protonated DPP, PDP and especially PPD visit also considerably different conformations. We note that an enhanced exploration of LHCSR1 at lower pH is found also in our CpHMD simulations (see Figure S5), although to a smaller extent. Interestingly, the drift in population shown by the CpHMD simulations agrees well with the exploration of PPD, i.e., as the pH is lowered, LHCSR1 explores analogous conformations to the ones observed in PPD. Other double protonated pMSs instead explore portions of tICA space that are not visited by the CpHMDs. Recalling also that the PPD population increases in the pH range associated with stress conditions (pH ∼ 5.5, Figure 4B), this is a further indication that this particular pMS explores structures potentially representative of quenched conformations.

As noted above, several pMSs can occupy the same region of tICA space; i.e., they present analogous conformations. On the other hand, we have seen that lowering the pH results in a higher exploration of tICA space; i.e., new free-energy minima in the LHCSR1 conformational landscape arise (see Figure 5A). In particular, doubly protonated pMSs show multiple minima in far apart regions of the tICA space, indicating that other stable conformations are present (Figure S4). As our primary goal is identifying quenched and unquenched conformations of LHCSR1, we are especially interested in characterizing these minima. To this end, we have employed the HDBSCAN^61^ clustering algorithm by using all of the replicas for all computed pMSs. This allows us to group together structures from each free-energy basin explored by our pMSs. The clustering is displayed in Figure 5B and the pMS composition of each cluster is presented in Figure 5C. More details on the clustering are provided in section S7.

We identified six clusters, well separated in the tICA space and covering regions associated with the minima of the reweighted free energy (see Figure S4). This indicates that our clusters are representative of the most stable structures for each pMS. Interestingly, as can be seen in Figure 5C, clusters CL1–CL4 are almost exclusively composed of the doubly protonated pMS (i.e., PDP, PPD and DPP), from which PPD and DPP are associated only with low pH conditions (Figure 4B). Clusters CL5 and CL6 are more heterogeneous, being composed of pMSs present at all the investigated pHs.

In order to visualize and analyze the structures that characterize the different clusters, we have extracted 30 random samples from each one, preserving the pMS composition of each cluster (Figure 5C). Figure 5D depicts a comparison between a representative structure of each cluster, selected as the one closest to the average structure, and the unbiased template (MD8). Notice that the selected visualization attempts to remark the structural similarities/differences of each cluster with respect to the starting MD8, making emphasis on helix D, helix E, and residues E114/E227/E233.

As observed in Figure 5D, in all the clusters, a remarkable conformational freedom is present in the lumenal part of LHCSR1, where the most evident effect is a strong rearrangement of both helix D and helix E. Close examination of the H-bond network in the triad E114, E227, and E233, revealed that such residues play a key role on the flexibility of helix D. Indeed, each cluster is characterized by a specific H-bond pattern involving two or all the residues in the triad. An additional effect is observed for CL2, where a conformational change of helix A appears at the level of the lumen. Helix A becomes distorted toward helix E, coupled to a conformational change of helix D. Such structural changes seem to be induced by a H-bond interaction between residues E227 and E233.

We are now interested in associating the clusters to pH by a comparison of the results reported in Figures 4B and 5. We focus on the two pH values (∼5.0 and 7.5) that have been previously shown to correspond to different conformations and to distinguish between quenched and unquenched states of the complex.^18,19^

At pH between 7 and 8, singly protonated pMS exist together with the doubly protonated structure PDP, while the contributions from all the other doubly protonated pMS are negligible (Figure 4). This heterogeneity is well represented by the CL5 conformation and less so by CL6 which contains a too large contribution from DPP and PPD. Lowering the pH to 6–5, the doubly protonated PPD becomes relevant, and this microstate explores a new region of the conformational space. This new conformation is the one represented by CL2, which is the only one composed almost entirely of PPD. Strikingly, the region of tICA space associated with CL2 is also considerably stable according to our free energy estimation of PPD (see Figure S4).

On the basis of these findings, in the following analysis, we consider CL2 as the conformation that becomes significant at pH ∼ 5.0, and that should represent the quenched state. On the other hand, as a representative conformation at pH ∼ 7.5, we consider CL5, as its composition is analogous to the pMS population at this pH (see Figure 4B). We also note that CL2 and CL5 are the ones with the largest number of frames (Table S2).

### 2.3 Quenching Mechanisms

In this section, we investigate how the LHCSR1 conformations associated with low pH (i.e., CL2) and high pH (i.e., CL5) conditions affect the EET and CT mechanisms of quenching. In particular, we focus our discussion on the putative quenching site based on the literature on LHCSR1 and other LHCs, i.e., dimer L1-Lut/*a*612.^19,35,62^

**Figure 6:**
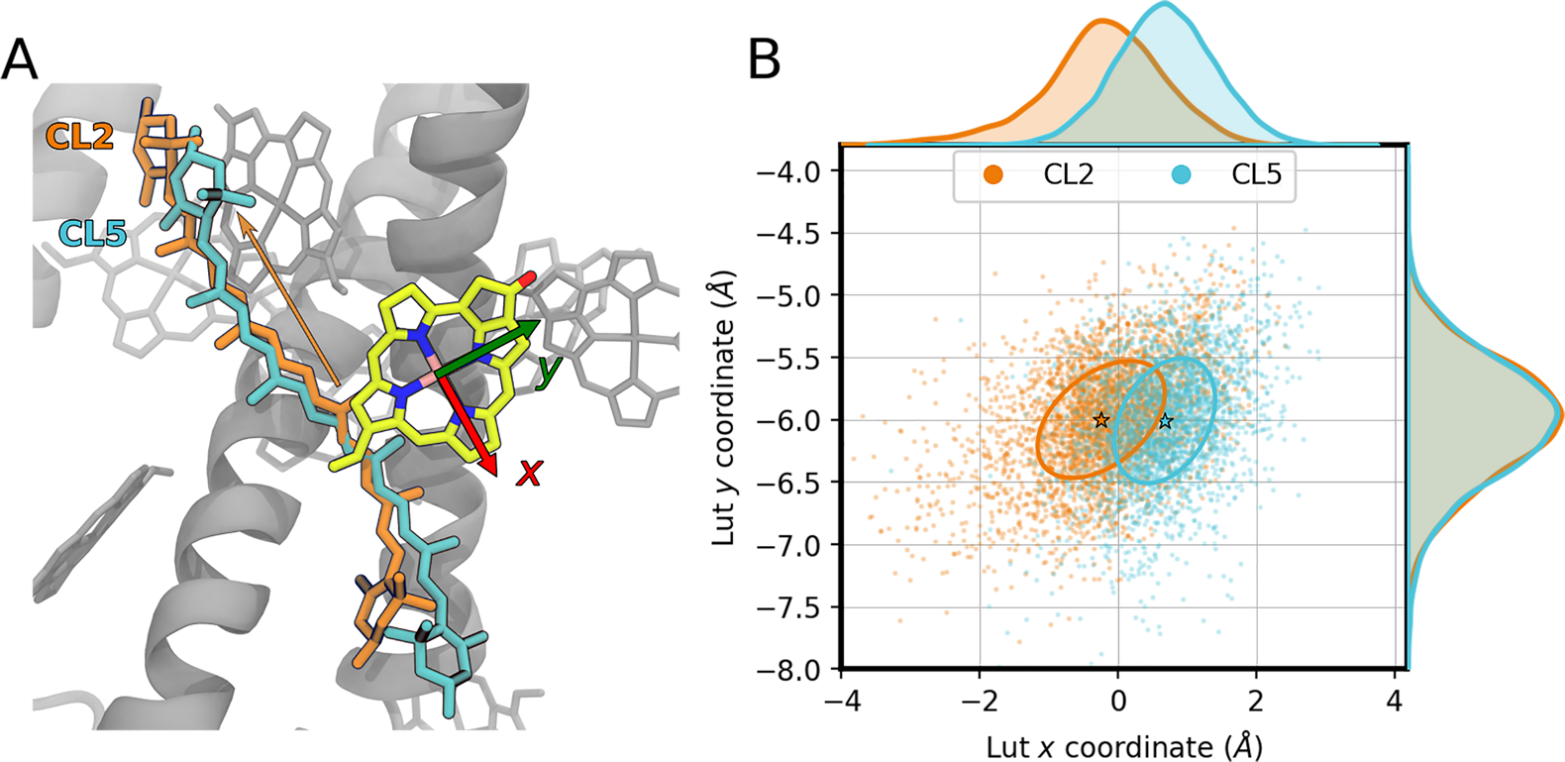
Geometrical analysis of the L1-Lut/Chl *a*612 pair in LHCSR1. (A) L1-Lut in a reference frame centered in Chl *a*612. The axes are colored red (*x*) and green (*y*). A representative structure from CL2 is shown alongside a representative structure from CL5. The orange arrow underlines the sliding toward the stroma of L1-Lut in CL2. (B) Scatter plot of the center of mass displacement of the L1-Lut isoprenic chain with respect to a reference system fixed onto the Mg^2+^ ion of Chl *a*612. The mean of each cluster is indicated with a star. The covariance ellipse enclosing 40% of the data is also reported.

As both energy and charge transfer processes are known to be sensitive to the intermolecular distances and orientations^30,63^ we have first analyzed the relative position of the two pigments in the two investigated clusters. Figure 6 represents the position of L1-Lut in a *xy* reference frame defined by the chlorin ring of *a*612 (the analogous analysis for all of the clusters is shown in Figure S9, section S11).

Remarkably, cluster CL2 presents a L1-Lut position significantly displaced along the *x* axis, slipping toward the stroma with respect to the center of mass of *a*612. As discussed above, the structural difference between cluster CL2 and CL5 mainly resides in the conformation of helix A (at the level of the lumen) and helix D: due to a H-bond interaction between E227(P) and E233(D), the C-terminus is more compact and closer to helix B. This has a direct effect not only on the arrangement of L1-Lut with respect to Chl *a*612, but also on the position of its terminal lumen-exposed ring, that is pushed toward helix B (see Figure 6A). Moreover, the lumenal loop is close to the core of the complex, making the overall structure of cluster CL2 more compact.

Overall, this analysis suggests that the protonation of lumen-exposed residues results in an altered geometry of the L1 site, which is expected to be reflected in the energy and charge transfer processes for the L1-Lut/*a*612 pair. This analysis is reported in the two following subsections.

#### 2.3.1 EET Quenching Mechanism

To investigate the EET mechanism, we computed EET couplings for the different conformations explored by our simulations using the TrEsp approximation^64^ (more details are provided in section 4.3). The results are reported in Figure S6. If we focus on the two main clusters (CL2 and CL5) we see that the largest Coulomb coupling, namely, that between L1-Lut and Chl *a*612, is insensitive to the geometrical differences of each clusters (this is also valid for the other clusters). This resilience is in line with the results recently obtained by us on CP29^65,66^ and shows that the non-negligible differences in the relative geometrical arrangement of L1-Lut and Chl *a*612 are not enough to significantly affect their EET coupling.

As the EET rate depends on both the coupling and the energy gap between the two involved excitations, we have further investigated the energy of the S_1_ state of L1-Lut by means of the semiempirical configuration interaction (SECI) approach, previously used with success for the description of the electronic structure of keto-carotenoids.^67,68^ Such calculations were carried out on the 30 representative structures of each cluster, in which L1-Lut was previously optimized at the DFT B3LYP/6-31G(d) level of theory in a QM/MM scheme, allowing the residues close to the L1-Lut to move (see details in section 4.3).

As it is well-known that the excitation energy of a carotenoid is affected by the internal geometry through a change of the degree of π-electron density delocalization, we have focused on an effective geometrical index, the so-called bond length alternation (BLA), namely, the difference between the average single and double bonds along the conjugated chain. Figure S7 reports the BLA and the S_1_ energies for the 30 representative structures of all the clusters. If we now focus on the two main clusters (CL2 and CL5) we see that there is a small difference in BLA but this does not correspond to a significant change in the S_1_ energy (Figure S7B).

From these results, we can conclude that the changes in the Lut-Chl interactions and Lut internal geometry induced by the change of the pH do not justify either a sizable variation in the couplings or in the excitation energy of the carotenoid. As a result, it appears that an EET channel at the L1 site cannot be tuned upon protonation of the lumenal residues in LHCSR1.

#### 2.3.2 CT Quenching Mechanism

We now turn to testing the possibility of a CT mechanism in the L1-Lut/*a*612 pair. The energies of the locally excited (LE) Chl Q_*y*_ and the charge transfer (CT) states, as well as their couplings, were determined using a multistate diabatization scheme^69^ on top of polarizable embedding QM/MM calculations (see section 4.3).

The computed average energies and couplings for the LE and CT states of clusters CL2 and CL5 are reported in Table 1. The complete data for all of the clusters are reported in Table S5. In all clusters, the lowest CT state corresponds to an electron transferred from L1-Lut to *a*612 (Lut^+^Chl^–^) and lies ca. 5000 cm^–1^ above the Q_*y*_ state of Chl*a*, in agreement with the results reported for both LHCII^30^ and CP29.^66^ Remarkably, although CP29 was initially used as a template for LHCSR1 homology modeling,^40^ the energy values of the Lut^+^Chl^–^ CT state obtained for LHCSR1 are more similar to LHCII (∼20200 cm^–1^)^30^ than CP29 (∼22500 cm^–1^).^66^ The lower energy of the Lut^+^Chl^–^ CT state in both LHCII and LHCSR1 can be explained in terms of an additional stabilization provided by a positively charged lysine, K179 in LHCII^30^ and K208 in LHCSR1,^40^ close to *a*612, which is replaced by an alanine in CP29,^66^ as illustrated in Figure S8 (see section S10). The average energy of the Lut^+^Chl^–^ CT state in LHCSR1 does not differ significantly between CL2 and CL5 (see Table 1), nor when considering all the other clusters (Table S5)

**Table 1:** Average Values and 95% Confidence Intervals of Energies of LE (Chl Q_*y*_) and CT (Lut^+^Chl^–^) States and LE-CT Couplings (V) in the L1-Lut/*a*612 Dimer for CL2 and CL5^a^

	Cluster	
Parameter	CL2	CL5
E(LE)	15582 ± 255	15689 ± 193
E(CT)	20656 ± 326	20887 ± 463
V(LE,CT)	292 ± 61	199 ± 46
*k*_*cs*_^b^	4.2	2.0
*k*_*cs*_^c^	14.6	6.8
*k*_*cs*_^d^	52.8	24.5
τ_complex_^b^	2929	3050
τ_complex_^c^	804	1121
τ_complex_^d^	148	285

a All values are in cm^–1^. Charge separation rates *k*_*cs*_(× 10^9^ s^–1^) were calculated using three different sets of reorganization energies (see section 4.4). Mean excitation lifetimes τ_*complex*_ (ps) of the LHCSR1 complex (section 4.5).

b Reorganization energies estimated for LHCSR1.

c Reorganization energies estimated for LHCII.^30^

d Reorganization energies estimated for CP29.^66^

Unlike energies, the charge-separation couplings, V(LE,CT), show a stronger dependence on the clusters, spanning from ∼200 (CL5) to ∼300 cm^–1^ (CL2). The V(LE,CT) couplings were finally used to compute the charge-separation rates (k_*CS*_) of the two clusters by applying the Marcus equation.^70^ A single effective Δ*G* was used for both clusters: its value was obtained by estimating the reorganization energies (λ), corresponding to the CT and LE state, in terms of the fluctuations of CT and LE vertical energies calculated along the MD simulations (see section 4.4).^71^ The use of this effective value is due to the large standard error obtained in the estimate of λ for the different clusters (Table S3). Due to this large uncertainty, we have tested two other sets of reorganization energies, namely, those previously calculated by us for two similar LHCs, LHCII,^30^ and CP29^66^ (see Table S4).

As can be seen, the obtained k_*CS*_ values (Table 1) strongly depend on the selected set of reorganization energies, which mainly differ in the value estimated for the CT state (see Table S5). However, for any choice of reorganization energy, the larger coupling in CL2 is reflected in a significantly larger *k*_*CS*_. By comparing the obtained results with the geometrical analysis of Figure 6, we can say that protonation of lumen-exposed residues results in an altered geometry of the L1 site with enhanced charge-separation rates for the L1-Lut/*a*612 pair. This finding shows that a CT quenching channel in LHCSR1 can indeed be modulated by the pH. These results are in line with the spectroscopic analyses on LHCSR1 in *Physcomitrella patens*(63) and on the similar LHCSR3 of *Chlamydomonas reinhardtii*(55,72) which have found evidence of a CT quenching mechanism.

Finally, we used the calculated k_*CS*_ rates in combination with a coarse-grained kinetic model^30,33^ to assess the impact of charge-transfer quenching on the excited-state lifetime of LHCSR1. This model features a pool of identical Chl *a*, with the exception of Chl *a*612 that can transfer energy to the CT state. Further details are given in section 4.5. The obtained lifetimes (Table 1) depend strongly on the employed set of *k*_*cs*_ values which, as commented before, are very sensitive to the value of reorganization energies. In fact, with the LHCSR1 set of parameters, we obtain lifetimes in the order of 2.9 ns for CL2 and 3.1 ns for CL5 which reduce to 0.8 and 1.1 ns, and to 148 and 285 ps, with reorganization energies estimated for LHCII and CP29, respectively. All these values are well within the lifetimes measured in single-molecule spectroscopy experiments on LHCSR1, which show a multicomponent dynamics corresponding to a lifetime distribution with two main peaks at <1 ns and 2–3 ns.^19^ Nonetheless, all three sets of lifetimes show that the differences between CL2 and CL5 are not large enough to fully explain the switch between a quenched and an unquenched state. It has, however, to be remarked that the calculated lifetimes are very sensitive to the value of the CT reorganization energies: differences of around 5% in the energy can shift the lifetime of the complex from subnanosecond to few nanosecond scales. Unfortunately, the present estimate of the reorganization energy from MD-based energy fluctuations is not robust enough to achieve such accuracy.

In light of this observation, it is likely that a more accurate estimate of the reorganization energy of the CT state could lead to a larger cluster-sensitivity. This is further supported by the significant difference found in the couplings of the two clusters due to the different dispositions of L1-Lut and Chl *a*612. We can thus suggest that the pH-induced conformation controls the quenching through an interplay of CT couplings and reorganization energies.

## 3 Conclusions

In this work, we investigated the conformational changes of LHCSR1 induced by pH-sensing in the thylakoid lumen. Combining constant-pH and enhanced sampling MD techniques, we sampled the conformational space of LHCSR1 by mimicking acidic and neutral pH conditions. Our analysis showed that conformations are explored at low pH that are not accessible at neutral pH, suggesting that a pH drop can exert a profound influence on the conformation of LHCSR1. More specifically, at low pH a much richer conformational landscape at the level of the lumen is explored by LHCSR1, whose variability is localized mainly on helix D and modulated by the coupled protonation states of three key residues, namely, E114, E227 and E233. In particular, we have identified a conformation that is well stabilized at low pH, and never explored at neutral pH, presenting a compact C-terminus closer to helix B. The conformational changes observed in this work could be further validated experimentally by point mutations on the aforementioned residues.

On the other hand, by performing QM/MM calculations, we found that the wide pH-induced conformational changes of the protein have a subtle effect on the lutein-chlorophyll interactions in the putative L1 quenching site. The EET couplings between Chls and Cars are marginally affected by the conformation and so is the energy of the S_1_ state of L1-Lut, suggesting that a EET quenching mechanism is not affected by pH changes. On the contrary, a CT-based quenching mechanism may be tuned by lowering lumenal pH, through conformational changes located near helix D inducing a displacement of lutein toward the stroma. This would provide a “soft switch” for increasing or decreasing the amount of quenching in LHCSR. On a final note, we also stress that multiple quenching pathways were detected in LHCSR proteins, and only some of them are modulated by pH variations.^55,63^ Indeed, some quenching pathways are activated or modulated by replacing Violaxanthin with Zeaxanthin at the L2 site.^19,73^ Therefore, a combination of different mechanisms may be necessary to fully explain the lifetime shortening in LHCSR1.

All of these findings strongly indicate that an investigation of the role of the aforementioned three residues in pH-sensitivity (e.g., via point mutations) and the simulation of the additional quenching pathways activated or modulated by replacing Violaxanthin with Zeaxanthin at the L2 site^19,73^ are required before a full understanding of the quenching in mosses can be reached.

## 4 Methods

### 4.1 Constant pH MD Simulations

We employed the so-called explicit-solvent CpHMD discrete approach, implemented by Roitberg and co-workers^41^ in the Amber18 package under the GPU-accelerated framework.^74^ Starting from the final structure of the 1 μs MD8 classical dynamics recently reported by some of us,^40^ a 5 ns short equilibration at pH 7.0 was carried out, restraining only the backbone of the transmembrane helices (A, B, C) by a 0.4 kcal mol^–1^ Å^–2^ harmonic restraint. Then, two independent sets of CpHMD production simulations were performed freely in the anisotropic NPT ensemble for each evaluated pH, ranging from 3.0 to 8.0 with a 1.0 spacing, for a total of 12 replicas. Each CpHMD replica was run for 600 ns for a total of 7.2 μs of simulation time. The Langevin thermostat and (for NPT simulations) the Monte Carlo anisotropic barostat were used to control temperature and pressure, respectively. The SHAKE algorithm was used in all simulations, allowing the use of a 2 fs integration time step. Long-range electrostatics were treated with the particle-mesh Ewald method, and a nonbonded cutoff of 10 Å was used. A distance restraint was included for the binding site Chl *a*613/Asn 226 to prevent chlorophyll detachment. The target residues were E114, D118, D126, E141, E149, E227, E232, and E233. Protonation state changes of these residues were attempted every 100 steps (200 fs), followed by 200 steps (400 fs) of solvent relaxation after a successful exchange. The other titratable groups were kept in their standard protonation state, except for Chl-binding histidines, which were δ-protonated with the aim of allowing Mg binding. For the Generalized Born calculations, a salt concentration of 0.1 was used. Further details are provided in section S1.

### 4.2 Gaussian Accelerated MD Simulations

We employed the GaMD module implemented in the Amber18 package under the GPU-accelerated framework^42^ for enhancing conformational sampling of LHCSR1 featuring six different pMSs. The parameters employed for MD simulations were similar to those specified above for CpHMD in section 4.1, except for the choice of an NVT ensemble. Each pMS was simulated by employing a GaMD protocol that uses dual-boost on both dihedral (σ_*D*_ = 6.0 kcal/mol) and nonbonded potential energy (σ_*P*_ = 6.0 kcal/mol) and sets the system threshold energy to the lower bound *E* = *V*_*max*_. The replicas were prepared as follows: starting from the template MD8 (see section 4.1), the protonation states of ionizable groups were fixed according to the studied pMS. More specifically, as illustrated in Figure 4A, each pMS is defined by either the protonated (P) or deprotonated (D) form of residues E114, E227, and E233. Moreover, in all cases, both E141 and E149 were protonated, Chl-binding histidines were δ-protonated, and the other ionizable residues were modeled in their standard protonation state. A 10 ns short conventional MD with no boost potential was performed to collect potential energy statistics for computing the GaMD accelation parameters, i.e., the maximum (*V*_*max*_), minimum (*V*_*min*_), average (*V*_*av*_), and standard deviation (σ_*V*_) of the system potential. Then, a 50 ns GaMD equilibration was run after adding the boost potential (ΔV). Finally, three independent sets of 2.0 μs GaMD production runs with randomized initial atomic velocities were performed, except for the case of pMS DPP where four replicas were run. The reweighted free energy profile of each pMS was obtained from cumulant expansion to the second order (section S5), by using the *PyReweighting*(75) toolkit. To assess the quality of the GaMD simulations, we monitored the boost potential (ΔV) along the simulations. The average (∼26.0 kcal mol^–1^) and standard deviation (∼6.8 kcal mol^–1^) of Δ*V* were found to be similar among all the pMSs (see Table S1 and Figure S3). These values ensure a good balance between exploration and stability of the simulation.^42^

### 4.3 QM/MM Calculations

The EET coupling between the L1-Lut(S_1_) and Chl *a*612(Q_*y*_) states was computed in the TrEsp approximation.^64^ Lut(S_1_) transition charges were taken from ref (76), where they were obtained from DFT/MRCI calculations. Chl *a*612(Q_*y*_) transition charges were obtained using TD-DFT B3LYP/6-31+G(d). Couplings were computed on a total of ca. 54000 frames in the six clusters extracted from our enhanced simulations (see Table S2). Couplings were rescaled by a factor of 3.7 in order to be comparable to previous work.^66,77^

A subset of representative frames (30 random samples) was extracted from each cluster by using a stratified sampling strategy that preserves pMS composition (see Figure S6). Geometry optimizations of the L1-Lut were performed on these structures, employing a QM/MM scheme. The QM subsystem containing the L1-Lut was described at the DFT/B3LYP/6-31G(d) level of theory. The MM layer was described with the same force field parameters used in the MD (section S1). MM residues within 6 Å of the QM subsystem were allowed to move during the optimization.

The optimized structures were employed to calculate the energy and properties of the L1-Lut S_1_ state. Semiempirical configuration interaction (SECI) calculations were run with the AM1 Hamiltonian, reparametrized for lutein on DFT and experimental data.^68^ We used the floating occupation molecular orbital (FOMO) framework,^78^ with a Gaussian width for floating occupation of 0.1 hartree, and we considered all the single and double excitations (CISD) in an active space of six electrons in nine molecular orbitals. The environment was included with electrostatic embedding QM/MM, with the MM layer described using the same force field parameters used in the MD simulations. The environment included all the residues within 30 Å from the lutein.

The optimized structures were also employed to obtain the energies of the LE and CT states of the L1-Lut/*a*612 pair as well as their couplings. For each structure, we computed the vertical energy of the first 10 excited states of the L1-Lut/*a*612 pair using a polarizable embedding QM/MM (QM/MMpol)^67^ in combination with the Tamm–Dancoff Approximation (TDA) formulation of TDDFT, using the ωB97X-D functional and the 6-31+G(d) basis set, and obtained the CT energies and LE/CT couplings with our multi-FED-FCD diabatization scheme.^69^ In the QM/MMPol calculations, the phytyl tail of the chlorophyll was cut after the first aliphatic carbon and included in the polarizable MM region. The energy of the Chl *a*612 Q_*y*_ (LE) excited state was recomputed with TD-DFT ωB97X-D/6-31+G(d), in order to be fully consistent with the analysis of ref (30). All QM/MMPol calculations were run using a locally modified version of Gaussian.^79^

### 4.4 Marcus Analysis for Charge-Separation Rates

The charge-separation rates (*k*_*cs*_) were computed by adopting the same protocol reported in ref (30). for LHCII. Briefly, it relies on the use of the Marcus rate equations for the estimation of the driving force and reorganization energy, based on the energy fluctuations of LE and CT states. Accordingly, the driving force *ΔG* = *G*_*CT*_ – *G*_*LE*_ for each cluster was obtained using the free energy of each state *X* (i.e., CT or LE) calculated in the linear response approximation^71^ as *G*_*X*_ = ⟨*E*_*X*_⟩ – λ_*X*_, where ⟨*E*_*X*_⟩ is the average energy of state *X*, and  with σ_*X*_^2^ being the variance of *E*_*X*_ estimated using 30 samples per cluster, and *k*_B_*T* the thermal energy. Then, an effective driving force was computed as the average value of the six clusters (Table S4). In parallel, an effective reorganization energy for the LE-CT process was obtained as  where σ_*CT,LE*_^2^ is the variance of *ΔE*_*CT,LE*_ estimated on the structures of all the clusters, i.e., 179 samples (see Table S4). A *k*_*cs*_ was then calculated for each cluster using the effective driving force and reorganization energy and the value of the coupling calculated for each cluster (see Table S5). Moreover, two further sets of *k*_*cs*_ were obtained by using reorganization energies estimated for LHCII^30^ and CP29.^66^ We note that the reorganization energies for CP29 differ slightly from those reported in ref (66) because here we have recomputed the Chl Q_*y*_ state in CP29 with TD-DFT, while in ref (66), we used the LE energies obtained from the diabatization. All values of the driving forces and reorganization energies are reported in Table S4.

### 4.5 Kinetic Model for Excitation Lifetime of the Complex

The mean excitation lifetime of the LHCSR1 complex (τ_*complex*_) was estimated for each of the six clusters, by adopting the same strategy used in ref (30) for the case of LHCII (see Table S5). Our model for LHCSR1 contemplates exclusively the site L1 to estimate excitation quenching. Chl *a*612 is assumed to be in fast equilibrium with the pool of the other seven Chls *a* and is able to transfer its population to the charge-separated state involving L1-Lut (i.e., *a*612*/L1-Lut^+^*a*612^–^). The charge-separated state can then recombine quickly (τ = 10 ps) to the ground state. Finally, to compute τ_*complex*_ we assume that the initial excitation is equally partitioned between all of the Chls *a* in the pool. To validate this assumption, we have repeated the analysis with an alternative model that includes explicitly site energies and couplings for all the eight Chls *a*. The obtained results, reported in section S13, confirm the validity of the coarse-grained model.

## Supporting Information Available

Details on molecular dynamics simulations; details on the fitting of CpHMD titration curves for p*K*_a_ estimation; protonation fraction of eight target ionizable residues; details about the protonation state transition analysis; raw data of acceleration parameters and free-energy reweighting for each GaMD replica; details on tICA construction and clustering; Coulomb couplings of selected Chls *a* with L1-Lut S_1_ state; L1-Lut BLA and S_1_ energy of each cluster; comparison between the L1 site of LHCSR1, LHCII, and CP29; L1-Lut position in the *a*612 reference frame for each cluster; raw data of the driving forces and reorganization energies for charge-separation of each cluster and average values; effect of Chl *a* site energies on the kinetic model for the lifetime of the complex; supplementary figures (PDF)
