# Supplementary material for: How the pH Controls Photoprotection in the Light-Harvesting Complex of Mosses
Source: J Am Chem Soc. 2023 Mar 24;145(13):7482–94. doi: 10.1021/jacs.3c00377 (PMC10080688; doi:10.1021/jacs.3c00377)
Supplement: Supplementary file 1 — ja3c00377_si_001.pdf [file ja3c00377_si_001.pdf]

Supporting Information: How the pH controls photoprotection in the light-harvesting complex of mosses

Laura Pedraza-González, Edoardo Cignoni, Jacopo D'Ascenzi, Lorenzo Cupellini, and Benedetta Mennucci*

Dipartimento di Chimica e Chimica Industriale, Università di Pisa, via G. Moruzzi 13, 56124 Pisa, Italy

E-mail: benedetta.mennucci@unipi.it

Contents

S1 Details on Molecular Dynamics simulations **S3**

S2 Fitting of CpHMD titration curves **S3**

S3 Protonation fraction for CpHMD target residues **S4**

S4 Protonation state transitions analysis **S6**

S5 GaMD acceleration parameters and reweighting **S6**

S6 tICA construction **S9**

S7 Clustering **S11**

S8 Coulomb couplings with Lutein L1 S1 state **S12**

S9 Bond Length Alternation and S1 Energy **S12**

S10 Comparison of the L1 site of LHCSR1, LHCII, and CP29 **S13**

S11 Lutein position in the a612 reference frame **S14**

S12 Reorganization energy and driving force **S15**

S13 Effect of Chl site energies on the kinetic model for the lifetime of the complex **S16**

S14 Supplementary Figures **S18**

References **S19**

S1 Details on Molecular Dynamics simulations

As mentioned in the main text, the sets of constant-pH (CpHMD) and Gaussian accelerated (GaMD) molecular dynamics simulations performed in this work started from a homology model of LHCSR1 recently reported by some of us.[S1] More specifically, we used as input structure the last frame of the 1 μs MD8 classical dynamics modeled in Ref.,[S1] which consists on a monomer of the protein-pigment complex embedded in a DOPC bilayer membrane and solvated with water molecules. For further details on the homology model construction we refer the user to the original publication.[S1] Moreover, the force fields employed for describing the protein, the pigments and the lipids are the same used for the homology model building. These are: the AMBER ff14SB force field for the protein; the force field by Prandi et al[S2] for the Carotenoids; the force field by Ceccarelli et al.[S3] with Zhang et al. modifications[S4] for chlorophylls *a*; the lipid14[S5] force-field for lipids. Water was described with the TIP3P model.

S2 Fitting of CpHMD titration curves

To obtain the calculated pK_a values and Hill coefficients (n) reported in Figure 2 in the main text, we fitted the protonated fraction along CpHMD simulations (f) and the pH to the modified Hill equation[S6–S8] (Eq. 1), by using the Levenberg-Marquardt nonlinear least-squares algorithm implemented in SciPy.[S9]

$$f = \frac{1}{1 + 10^{n(pH - pKa)}} \tag{1}$$

S3 Protonation fraction for CpHMD target residues

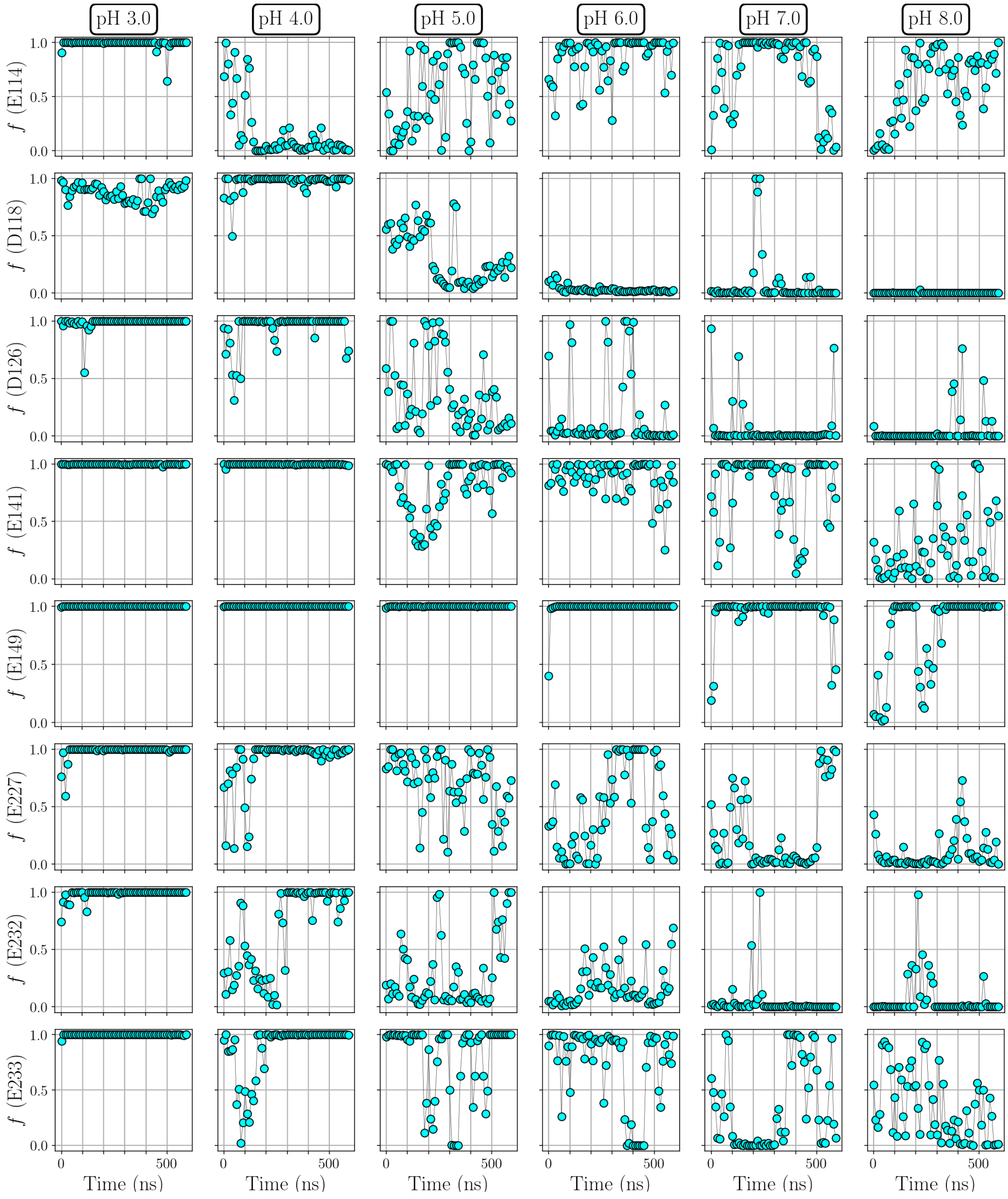

Figure S1: Protonation fraction (f) for eight lumenal residues in LHCSR1. Each point represent the average value of a chunk of 10 ns. Data correspond to the **first set** of CpHMD replicas performed at pH 3.0 to 8.0.

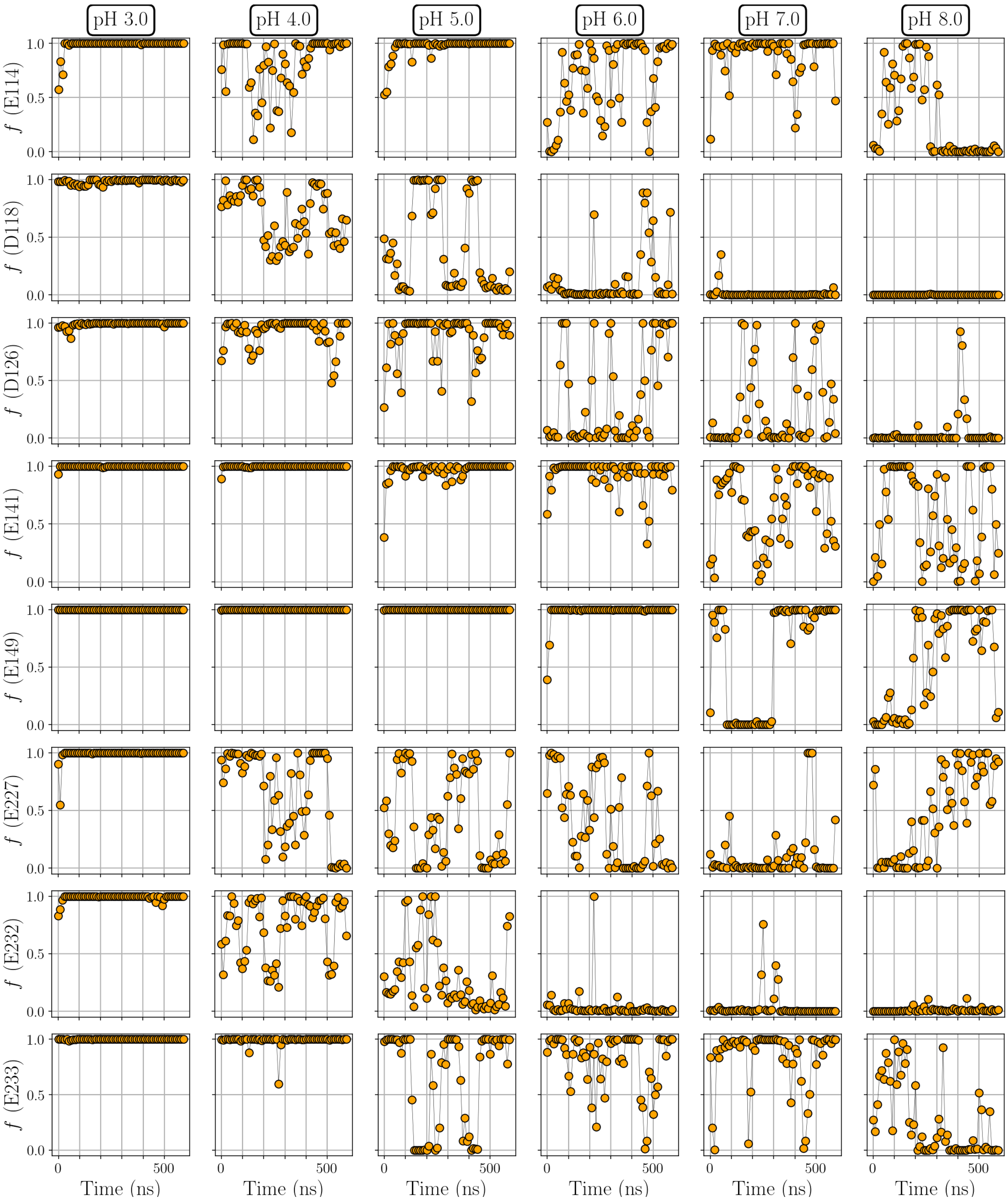

Figure S2: Protonation fraction (f) for eight lumenal residues in LHCSR1. Each point represent the average value of a chunk of 10 ns. Data correspond to the **second set** of CpHMD replicas performed at pH 3.0 to 8.0.

S4 Protonation state transitions analysis

In order to analyze the protonation patterns sampled during the constant pH MD, we have constructed a network model in a similar spirit to what is done in Ref. S10. First, starting from the protonation state trajectories visited during the constant pH simulations, we have mapped each frame to a discrete index identifying a protonation microstate. The protonation microstates that we have considered are all the possible combinations (eight) of protonated/deprotonated residues formed by the triad E114-E227-E233 (using P for protonated and D for deprotonated, these microstates are PPP, PPD, PDP, DPP, DDP, DPD, PDD, DDD).

These mapped (featurized) trajectories have been employed to build a Markov matrix $\mathbf{T}$, where $\mathbf{T}_{ij} = p_{ij}$ is the probability of going from microstate i to microstate j. This matrix was constructed by counting transitions from the i-th microstate to the j-th microstate using the "sliding window" scheme and employing a maximum likelihood Markov model estimation.[S11] This matrix was finally employed to visualize the transitions and the stability of each microstate with the aid of the `pyEMMA`[S12] software, as shown in Figure 4 in the main text. In this plot, the circle sizes are proportional to the entries of the stationary distribution π of the transition matrix, $\pi = \mathbf{T}\pi$. The arrows show the fluxes $f_{ij} = \pi_i p_{ij}$ associated with the transition from i to j.

S5 GaMD acceleration parameters and reweighting

Since a comprehensive review about the methodological framework and applications of the Gaussian Accelerated Molecular Dynamics (GaMD) technique is provided in Ref. S13, in this work we only specify the computational details needed to reproduce our reported results (see Section 4.2 in the main text).

In order to obtain an accurate reweighting of the free energy profile for each pMS by using the cumulant expansion to the second order, acceleration parameters of the applied boost potential (ΔV) were collected every 2 ps along each of the 2.0 μs GaMD replicas, for

a total of c.a. $10x10^6$ data. Therefore, c.a. $30x10^6$ values of ΔV for each pMS were used for reweighting, except for DPP that used c.a. $40x10^6$. Figure S3 illustrates the gaussian distribution of these data, Table S1 reports the statistics of ΔV in terms of average and standard deviation per pMS, and Figure S4 shows the 2D reweighting of the free energy projected onto our tICA space.

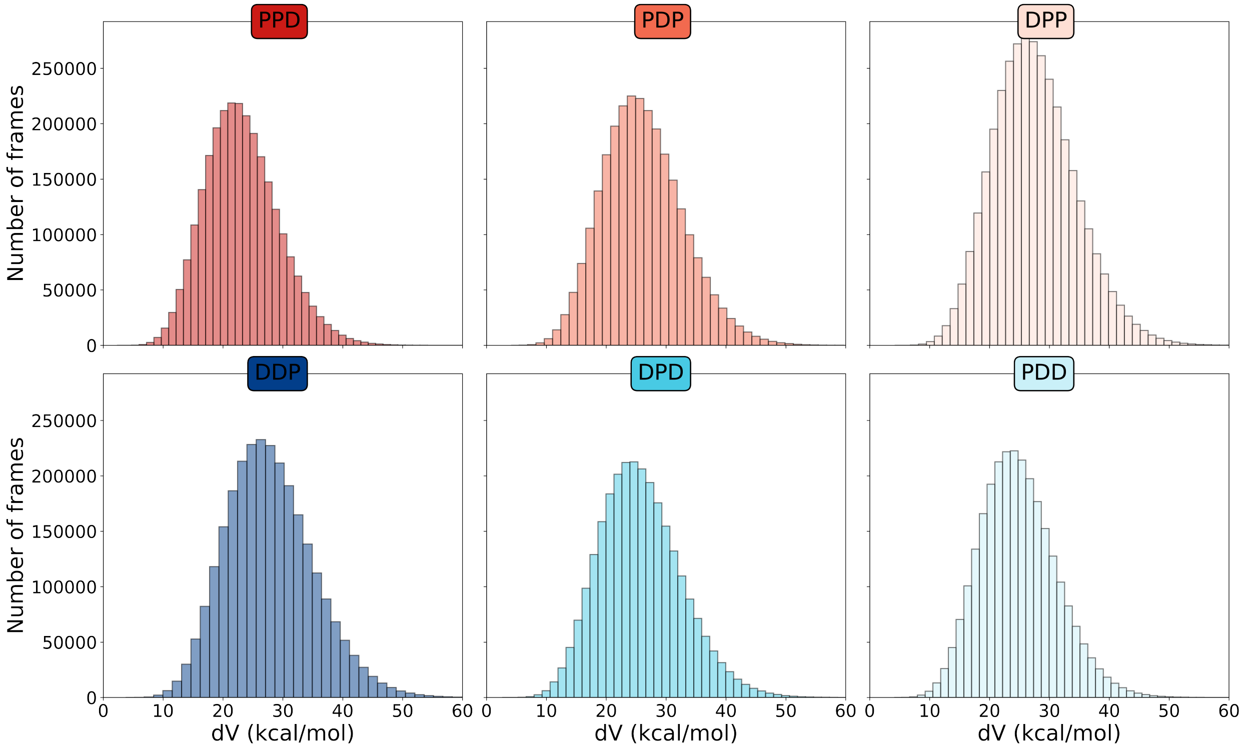

Figure S3: Gaussian distribution of the boost potential applied to the GaMD simulations performed for six pMSs. Each plot represent compiled data of three independent replicas of 2.0 μs each, except DPP which contains four replicas.

Table S1: Boost potential (ΔV) applied in GaMD simulations of six protonation microstates of LHCSR1. Data is presented as the average and standard deviation (σ) values of three independent replicas of 2.0 μs each, except DPP which contains four replicas.

	GaMD	ΔV (kcal mol^{-1})	
pMS	length, μs	Avg	σ
PPD	6.0	23.17	6.14
PDP	6.0	26.14	6.74
DPP	8.0	27.56	6.91
DDP	6.0	27.85	7.32
DPD	6.0	25.72	6.82
PDD	6.0	24.85	6.32

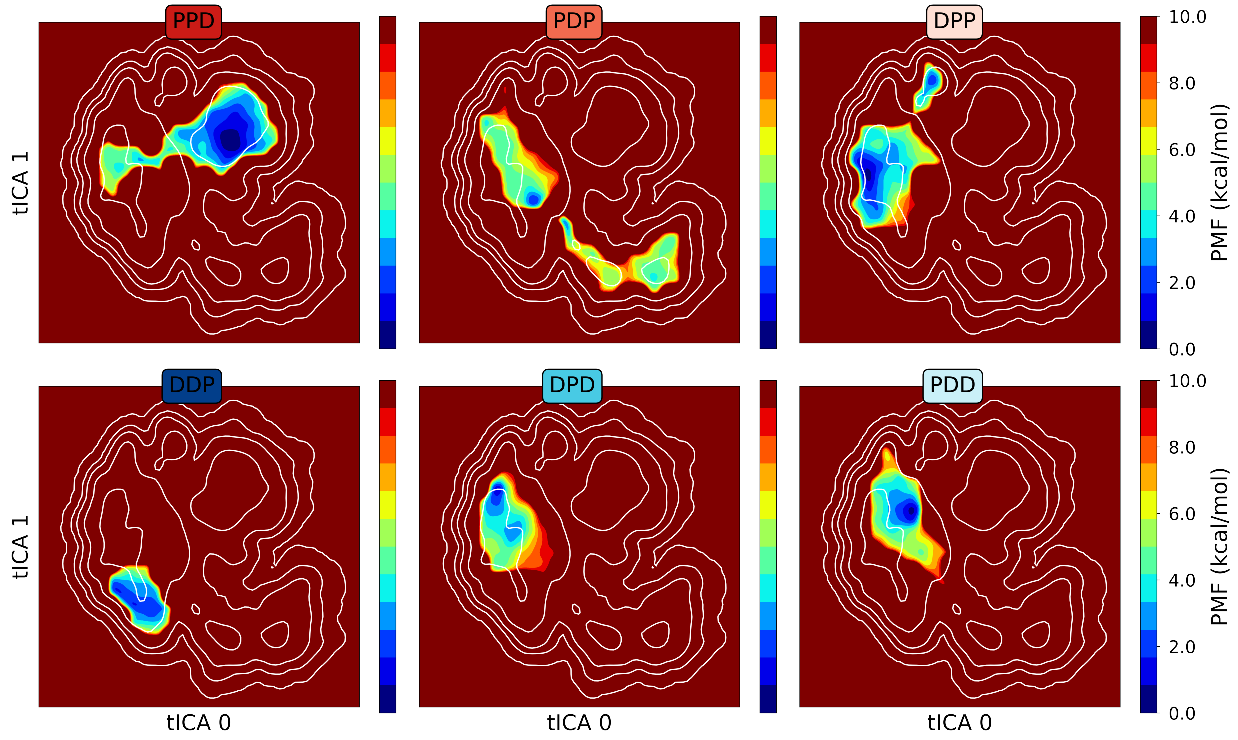

Figure S4: 2D PMF (tICA 0, tICA 1) profile of each of the six analyzed pMSs, calculated by reweighting the set of 2.0 μs GaMD replicas. The first 200 ns of each replica were excluded from reweighting. The white contour represents the tICA reduced space (see Section S6).

S6 tICA construction

In order to identify the slow modes that drive the enhancing conformational sampling in our set of GaMD simulations, which mimic how the conformational space explored by LHCSR1 is modulated by specific pH-dependent protonation microstates (pMS), we have employed the time-lagged Independent Component Analysis (tICA). Such an approach is a dimensionality reduction method widely used for the interpretation of structures obtained from molecular dynamics, which allows linearly transforming a predefined set of structural parameters (*e.g.* distances, angles, and dihedrals) into collective coordinates sorted by "slowness", to get a two-dimensional description of the conformations.[S14–S16] As further described in the main text, we concentrate our analysis on the interpretation of structural changes evidenced in the lumenal side of LHCSR1, by selecting distances and side chain dihedrals involving the three residues characterizing the pMS (E114, E227, and E233), as well as distances and angles describing the relative positions of helices A, B, D, and E. The selected 17 structural parameters are listed as follows:

Distances

1. E114@CD - E227@CD,
2. E227@CD - E233@CD,
3. E114@CD - E233@CD,
4. E114@CA - P235@CA,
5. Y123@CA - S230@CA,
6. E114@CA - R241@CA,
7. W123-D126@CA - T98-R101:I155-C158@CA,
8. A103@CA - S130@CA,
9. E114@CA - P137@CA,

Angles

10. E114@CA - E227@CA - P235@CA,
11. Q226@CA - I234@CA - R241@CA,
12. A200@CA - Q226@CA - R241@CA,
13. A103@CA - I129@CA - L152@CA,

Side-chain dihedrals

14. sin χ_1-E233,
15. cos χ_1-E233,
16. sin χ_2-E233,
17. cos χ_2-E233

The above listed parameters were computed for 90000 structures (*i.e.*, frames) from each of the 19 GaMD replicas, extracted using an equidistant time step along the 2.0 μs of simulation and after excluding the first 200 ns. Therefore, structural data of a total of 171000 frames was used as input for training the tICA algorithm implemented in the Deeptime python library,[S17] using a lagtime of 2 ns. As a result, the two coordinates that show the greatest correlation time was selected as the first two tICA eigenvectors (tICA1 and tICA2) defining our tICA space. As shown in Figure 5A in the main text, such space was used to project not only the six studied pMSs, but also the unbiased MD8 that was not included in the training set. Moreover, Figure S5 plots the projection of the set of CpHMDs.

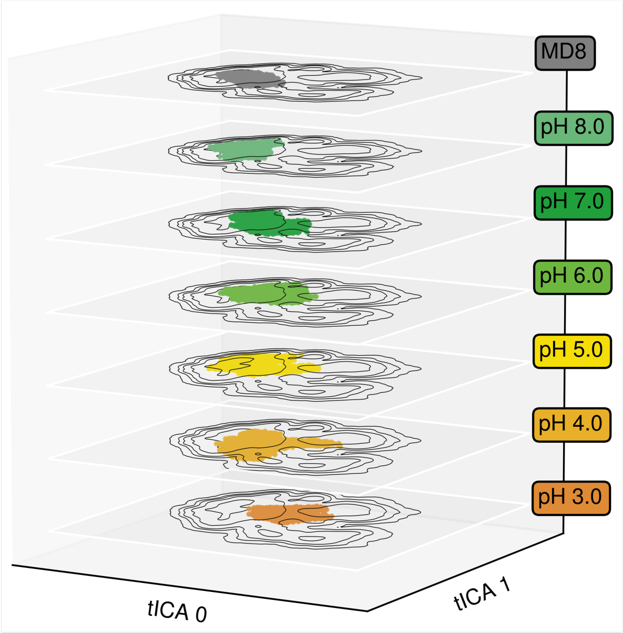

Figure S5: Projection of CpHMDs onto the tICA space. Individual CpHMD simulations for each pH are projected onto the first two principal tICA components computed for the GaMD simulations on pMSs. The first 100 ns of each MD were excluded from the calculation of structural parameters.

S7 Clustering

The tICA space determined from our featurization of the accelerated simulations was used as a starting point for the definition of clusters of structures similar to each other. The clustering was performed using the Python implementation of hdbscan,[S18] a density-based clustering algorithm. The hyperparameters of the model, namely `min_samples`, `min_cluster_size`, and `cluster_selection_epsilon`, were optimized with the aid of `scikit-learn`[S19] by performing cross-validation (CV) over a random search in hyperparameter space. As a CV score to be maximized, we have chosen the validity index[S20] as provided by the hdbscan software. The validity index is higher for dense clusters separated by regions of low density, and therefore favors the choice of small and densely populated clusters. Among the set of hyperparameters yielding the highest CV score, we have chosen the one that provided the more evenly-sized clusters. Finally, the structures from the learned clusters were visualized with VMD,[S21] and one cluster which showed significantly heterogeneous interactions of residues E114, E227, E233 was manually split in two. This procedure finally yielded six clusters. We note that not all structures belong to a cluster, as the sparsely populated regions of the tICA space are assigned to noise.

Table S2: Number of frames included in each cluster

CL1	CL2	CL3	CL4	CL5	CL6
2076	15928	2401	2064	13228	18142

S8 Coulomb couplings with Lutein L1 S1 state

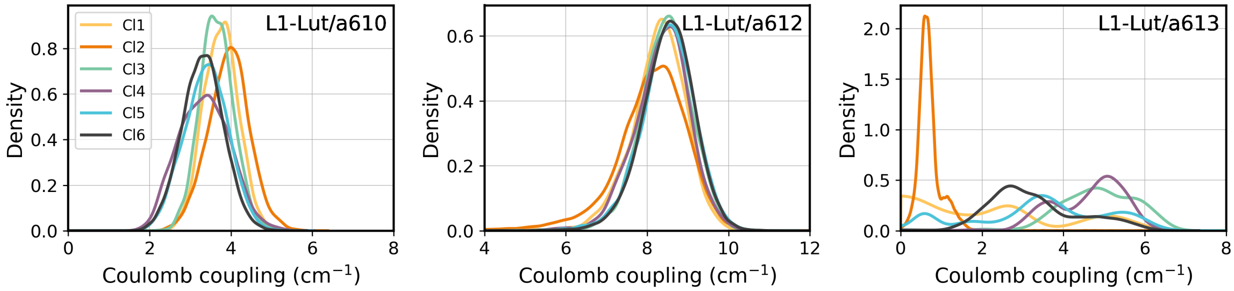

Figure S6: Coulomb couplings between the Lutein (LutL1) S_1 state and the chlorophyll Q_y state. Only the three chlorophylls most coupled to LutL1 are shown. The distributions are shown for the absolute value of the coupling.

S9 Bond Length Alternation and S1 Energy

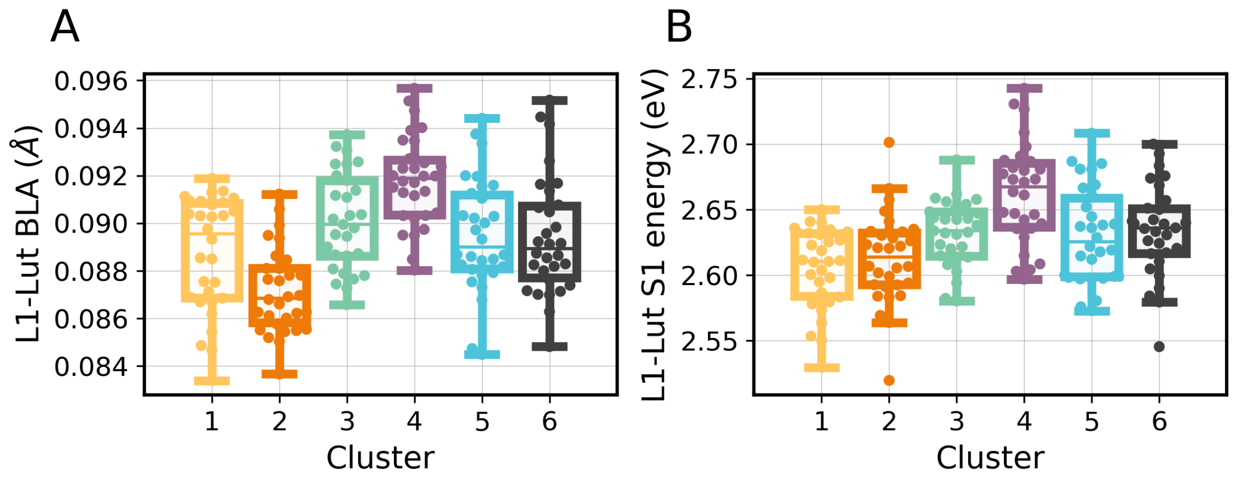

Figure S7: Bond Length Alternation (BLA) and S_1 energy for Lutein in the L1 site of LHCSR1. A. Bond Length Alternation computed along the conjugated chain of Lutein. B. Excitation energy (vertical energy) of the S_1 state of Lutein, as computed with semiempirical CISD (details on the semiempirical calculations are provided in the main text).

S10 Comparison of the L1 site of LHCSR1, LHCII, and CP29

In the main manuscript, we show that the energy of the CT state $Lut^{+}Chl^{-}$ in LHCSR1 is similar to that in LHCII,[S22] but different from the one we have obtained in CP29 for the analogous Car-Chl pair.[S23] This stabilization of the CT state was initially rationalized in Ref. S22 in terms of the presence of a positively charged lysine residue (Lys179). Indeed, an analysis of the residues in the L1 pocket of LHCSR1 shows that an analogous lysine residue, Lys208, is present and accounts for the stabilization of the CT state. CP29 does not show the lysine nor a different positively charged residue. The presence/absence of the lysine is shown in Figure S8.

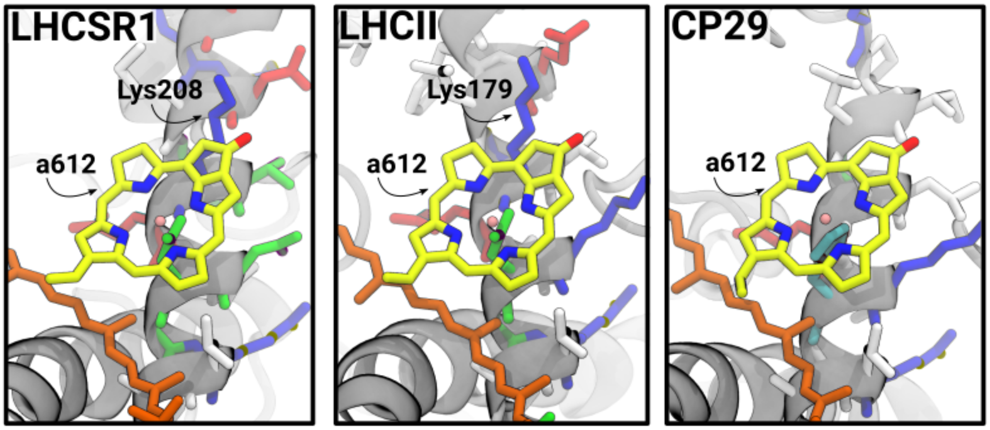

Figure S8: Comparison of the residues in the L1 site of LHCSR1 (left), LHCII (center), and CP29 (right). In the three cases, the image is centered on the Chl a612, taken as a common reference. LHCSR1 and LHCII have a lysine residue near the Lutein-Chl pair. CP29, on the other hand, does not present the lysine nor an analogous positively charged residue. Residues along helix A are colored according to the residue type: white for non-polar, blue for basic, red for acidic, and green for polar residues.

S11 Lutein position in the $a612$ reference frame

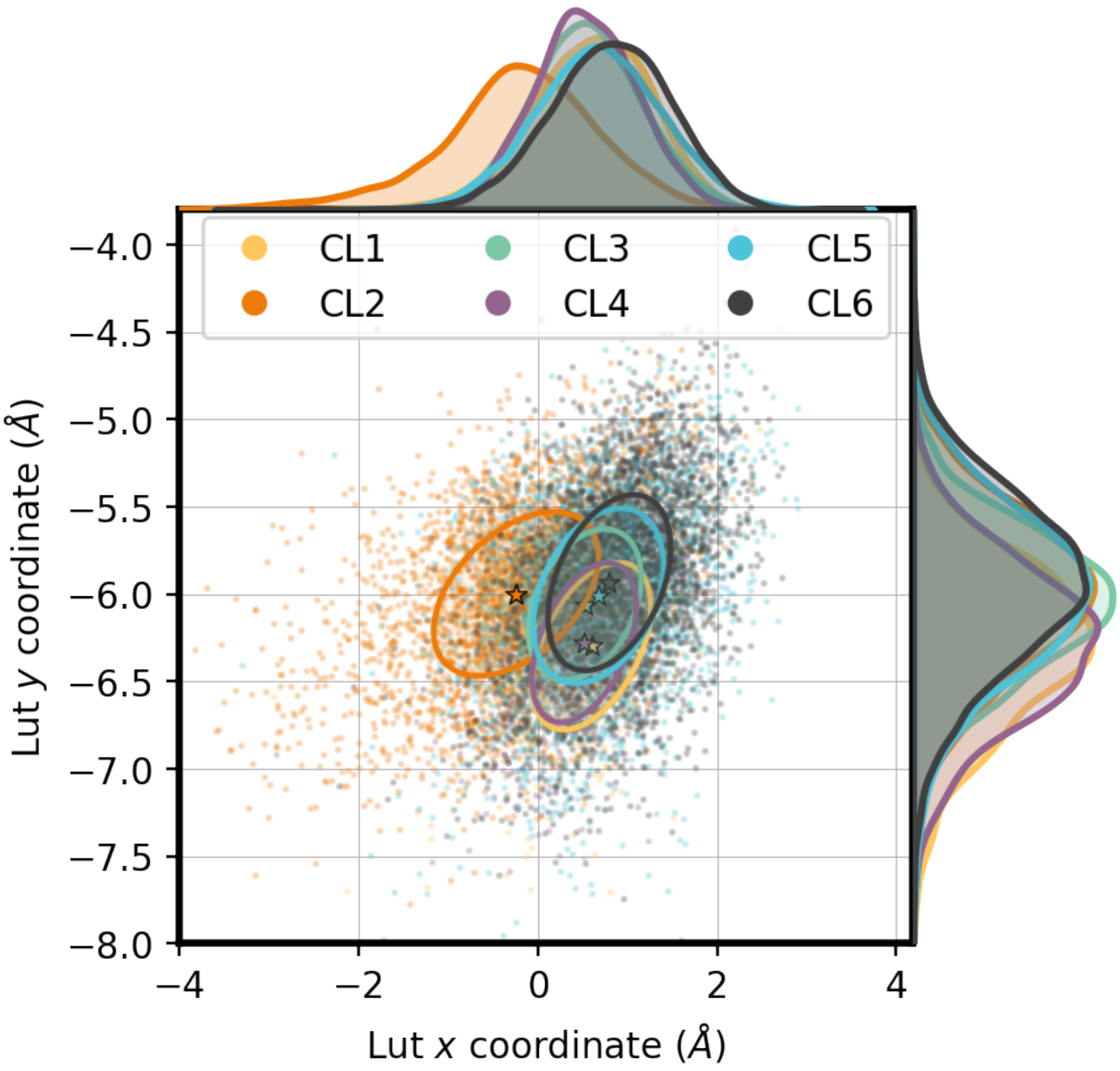

Figure S9: Scatter plot of the center of mass displacement of the L1-Lut isoprenic chain with respect to a reference system fixed onto the Mg^{2+} ion of Chl $a612$. The mean of each cluster is indicated with a star. The covariance ellipse enclosing 40% of the data is also reported.

S12 Reorganization energy and driving force

Table S3: Reorganization energies (λ) of the pair L1-Lut/a612 in LHCSR1, estimated from the variance of the CT and LE energies of each cluster. All values are in cm^{-1}. Bootstrap standard error (1000 bootstrapped samples) are also presented.

	Cluster						ALL
	CL1	CL2	CL3	CL4	CL5	CL6	
Samples	30	30	29	30	30	30	179
λ_{CT}	4388 ± 926	4677 ± 1282	5774 ± 1313	4680 ± 1267	5845 ± 1365	3650 ± 962	4919 ± 520
λ_{LE}	1115 ± 243	873 ± 173	703 ± 138	588 ± 188	640 ± 113	737 ± 201	773 ± 76
λ_{CTLE}	5106 ± 1118	4374 ± 1082	5250 ± 1341	5481 ± 1309	5754 ± 1111	4212± 1128	5082 ± 510

Table S4: Reorganization energies (λ) and driving forces (ΔG), of the pair L1-Lut/a612 in LHCSR1, estimated from the variance of the CT and LE energies. Reported data for LHCII[S22] and CP29[S23] is also provided. Values for CP29 are obtained with the same data computed in Ref. S23, except for the LE excitation energy, which is computed with the full TD-DFT as in Ref. S22, in order to be fully consistent. All values are in cm^{-1}.

Parameter	LHCII[S22]	CP29[S23]	LHCSR1
Samples	240	300	179
λ_{LE}	770	831	773
λ_{CT}	5520	6489	4919
λ_{CTLE}	5405	6091	5082
ΔG_{CTLE}	415	-493	1019

Table S5: Average values and 95% confidence intervals of energies of LE (Chl Q_y) and CT (Lut^+Chl^-) states and LE-CT couplings (V) in the L1-Lut/a612 dimer. All values are in cm^{-1}. Charge recombination rates k_{cs}(x 10^9 s^{-1}) calculated using three different sets of reorganization energies (see Methods Section 4.4).

	Cluster					
Parameter	CL1	CL2	CL3	CL4	CL5	CL6
E(LE)	15628 ± 255	15582 ± 225	15491 ± 206	15495 ± 185	15689 ± 193	15444 ± 207
E(CT)	20665 ± 345	20656 ± 326	20881 ± 391	20579 ± 289	20887 ± 463	20662 ± 270
V(LE,CT)	164 ± 40	292 ± 61	223 ± 57	212 ± 39	199 ± 46	186 ± 52
k^a_{cs}	1.3	4.2	2.5	2.2	2.0	1.7
k^b_{cs}	4.6	14.6	8.5	7.7	6.8	5.9
k^c_{cs}	16.6	52.8	30.8	27.8	24.5	21.5
$\tau^a_{complex}$ (ps)	3139	2929	3008	3026	3050	3078
$\tau^b_{complex}$ (ps)	1361	804	1008	1056	1121	1197
$\tau^c_{complex}$ (ps)	401	148	234	255	285	320

Reorganization energies estimated for ^{a}LHCSR1, ^{b}LHCII,[S22] and ^{c}CP29[S23] .

S13 Effect of Chl site energies on the kinetic model for the lifetime of the complex

As specified in Section 4.5 in the main text, the coarse-grained kinetic model employed in this work makes a series of approximations that might affect the computed rates and complex lifetimes. For instance, it considers exclusively the CT channel L1-Lut/*a*612 (charge-separate state a612*/L1-Lut$^+$*a*612), and assumes that *a*612 is in fast equilibrium with the pool of the other seven Chls *a* present in LHCSR1 (see Figure S11). The latter represents a strong assumption since it equally populates the *a* Chls in the pool. (see Section 4.3).

To validate this assumption, we used an alternative model[S24] that explicitly includes site energies and couplings for the eight Chl *a*. With this new model we also account for exciton delocalization among closely coupled *a* Chls, previously identified by Guarnetti Prandi et al. based on the exciton structure of LHSCR1, *i.e.*, excitonic domains *a*603-*a*609 and *a*610-*a*611-*a*612. We generalized the exciton Hamiltonian matrix obtained in Ref. S1 for LE states only to include the L1-Lut/*a*612 CT state using CT energies and CT-LE couplings reported in Table S5. To be consistent with the LE energies calculated in Ref. S1 the CT energy was determined by adding to the energy of *a*612 the CT-LE average energy difference reported in Table S5. After building the generalized exciton Hamiltonian, we performed a block diagonalization of the exciton domains as described in Ref.[S24] Then, by using each of the three sets of reorganization energies listed in Table S4 we computed the driving force (ΔG) and rates for each Chl *a* exciton in the *a*610-*a*611-*a*612 domain. Finally, we computed the mean excitation lifetime of the complex.

The results are presented in Table S6 and Figure S10. As observed, the new lifetimes have the same order of magnitude of those obtained with the coarse-grained model (see Table S5) when comparing values obtained with the same set of reorganization energies. Interestingly, as illustrated in Figure S10, both models produce the same trend in $\tau_{complex}$ among the clusters. We conclude therefore that improving the description of the Chl part

in the model does not alter the results obtained with the coarse grained model reported in the main text.

Table S6: Mean excitation lifetime ($\tau_{complex}$) of the LHSCR1 complex, computed for each cluster with the all-Chls *a* kinetic model. Values are computed with three sets of reorganization energies.

	CL1	CL2	CL3	CL4	CL5	CL6
$\tau^{a}_{complex}$ (ps)	3526	3396	3446	3457	3472	3490
$\tau^{b}_{complex}$ (ps)	2035	1350	1620	1682	1762	1850
$\tau^{c}_{complex}$ (ps)	711	271	427	466	517	577

[a] Reorganization energies estimated for LHCSR1 .

[b] Reorganization energies estimated for LHCII.[S22]

[c] Reorganization energies estimated for CP29.[S23]

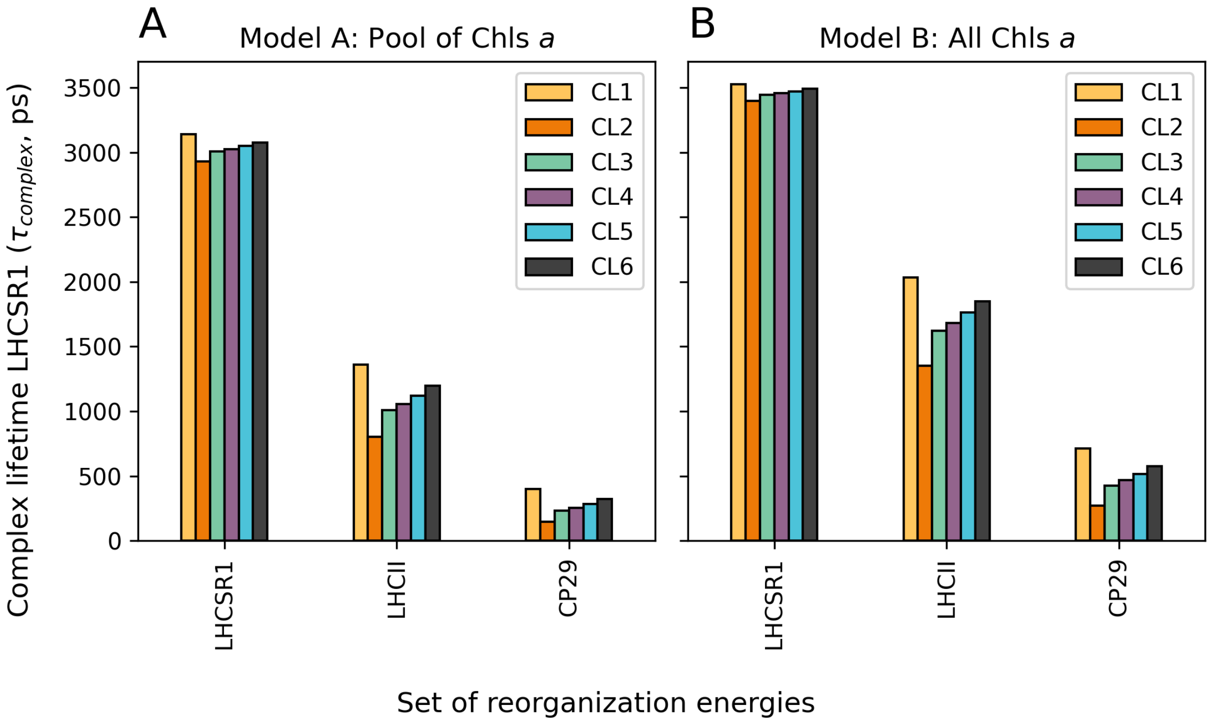

Figure S10: Mean excitation lifetimes $\tau_{complex}$ of the LHSCR1 complex. A. Kinetic coarse-grained model that assumes that $a612$ is in fast equilibrium with the pool of the other seven Chls *a* present in LHCSR1. B. Kinetic model that includes the effect of each Chl *a* and excitonic domanis in the complex. Values are computed with three sets of reorganization energies.

S14 Supplementary Figures

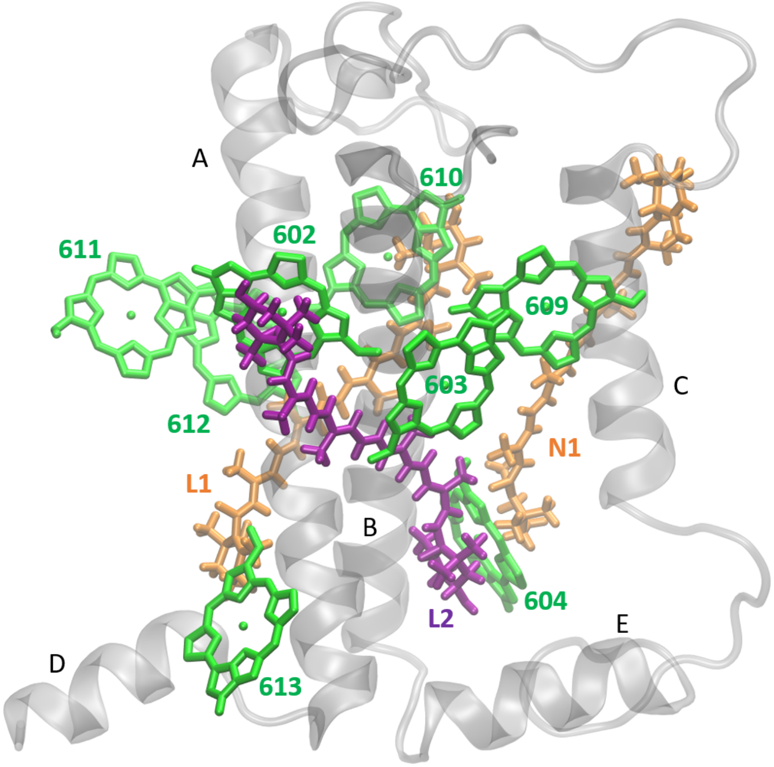

Figure S11: Pigments composition in *Pp*LHCSR1. The complex contains 8 chlorophylls *a* (green), two luteins located in the sites called *L1* and *N1* (orange), and a violaxanthin (purple) in the *L2* site.

References

(S1) Guarnetti Prandi, I.; Sláma, V.; Pecorilla, C.; Cupellini, L.; Mennucci, B. Structure of the stress-related LHCSR1 complex determined by an integrated computational strategy. *Commun. Biol.* **2022**, *5*, 1–10.

(S2) Prandi, I. G.; Viani, L.; Andreussi, O.; Mennucci, B. Combining classical molecular dynamics and quantum mechanical methods for the description of electronic excitations: The case of carotenoids. *J. Comput. Chem.* **2016**, *37*, 981–991.

(S3) Ceccarelli, M.; Procacci, P.; Marchi, M. An ab initio force field for the cofactors of bacterial photosynthesis. *J. Comput. Chem.* **2003**, *24*, 129–142.

(S4) Zhang, L.; Silva, D.-A.; Yan, Y.; Huang, X. Force field development for cofactors in the photosystem II. *J. Comput. Chem.* **2012**, *33*, 1969–1980.

(S5) Dickson, C. J.; Madej, B. D.; Skjevik, Å. A.; Betz, R. M.; Teigen, K.; Gould, I. R.; Walker, R. C. Lipid14: the amber lipid force field. *J. Chem. Theory Comput.* **2014**, *10*, 865–879.

(S6) Onufriev, A.; Case, D. A.; Ullmann, G. M. A Novel View of pH Titration in Biomolecules. *Biochemistry* **2001**, *40*, 3413–3419.

(S7) Machuqueiro, M.; Baptista, A. M. Acidic range titration of HEWL using a constant-pH molecular dynamics method. *Proteins* **2008**, *72*, 289–298.

(S8) Williams, S. L.; Blachly, P. G.; McCammon, J. A. Measuring the successes and deficiencies of constant pH molecular dynamics: A blind prediction study. *Proteins* **2011**, *79*, 3381–3388.

(S9) Virtanen, P.; Gommers, R.; Oliphant, T. E.; Haberland, M.; Reddy, T.; Cournapeau, D.; Burovski, E.; Peterson, P.; Weckesser, W.; Bright, J.; van der Walt, S. J.; Brett, M.; Wilson, J.; Millman, K. J.; Mayorov, N.; Nelson, A. R. J.; Jones, E.;

Kern, R.; Larson, E.; Carey, C. J.; Polat, İ.; Feng, Y.; Moore, E. W.; VanderPlas, J.; Laxalde, D.; Perktold, J.; Cimrman, R.; Henriksen, I.; Quintero, E. A.; Harris, C. R.; Archibald, A. M.; Ribeiro, A. H.; Pedregosa, F.; van Mulbregt, P.; SciPy 1.0 Contributors, SciPy 1.0: Fundamental Algorithms for Scientific Computing in Python. *Nat. Methods* **2020**, *17*, 261–272.

(S10) Hofer, F.; Dietrich, V.; Kamenik, A. S.; Tollinger, M.; Liedl, K. R. pH-Dependent Protonation of the Phl p 6 Pollen Allergen Studied by NMR and cpH-aMD. *J. Chem. Theory Comput.* **2019**, *15*, 5716–5726.

(S11) Bowman, G. R., Pande, V. S., Noé, F., Eds. *An Introduction to Markov State Models and Their Application to Long Timescale Molecular Simulation*; Springer Netherlands, 2014.

(S12) Scherer, M. K.; Trendelkamp-Schroer, B.; Paul, F.; Pérez-Hernández, G.; Hoffmann, M.; Plattner, N.; Wehmeyer, C.; Prinz, J.-H.; Noé, F. PyEMMA 2: A Software Package for Estimation, Validation, and Analysis of Markov Models. *J. Chem. Theory Comput.* **2015**, *11*, 5525–5542.

(S13) Wang, J.; Arantes, P. R.; Bhattarai, A.; Hsu, R. V.; Pawnikar, S.; Huang, Y.-m. M.; Palermo, G.; Miao, Y. Gaussian accelerated molecular dynamics: Principles and applications. *Wiley Interdiscip. Rev.: Comput. Mol. Sci.* **2021**, *11*, e1521.

(S14) Naritomi, Y.; Fuchigami, S. Slow dynamics in protein fluctuations revealed by time-structure based independent component analysis: the case of domain motions. *J. Chem. Phys.* **2011**, *134*, 065101.

(S15) Pérez-Hernández, G.; Paul, F.; Giorgino, T.; De Fabritiis, G.; Noé, F. Identification of slow molecular order parameters for Markov model construction. *J. Chem. Phys.* **2013**, *139*, 015102.

(S16) Schwantes, C. R.; Pande, V. S. Improvements in Markov State Model Construction Reveal Many Non-Native Interactions in the Folding of NTL9. *J. Chem. Theory Comput.* **2013**, *9*, 2000–2009.

(S17) Hoffmann, M.; Scherer, M.; Hempel, T.; Mardt, A.; de Silva, B.; Husic, E. B.; Klus, S.; Wu, H.; Kutz, N.; Brunton, L. S.; Noé, F. Deeptime: a Python library for machine learning dynamical models from time series data. *Mach. learn.: sci. technol.* **2021**, *3*, DOI: 10.1088/2632–2153/ac3de0.

(S18) McInnes, L.; Healy, J.; Astels, S. hdbscan: Hierarchical density based clustering. *J. Open Source Software* **2017**, *2*, 205.

(S19) Pedregosa, F.; Varoquaux, G.; Gramfort, A.; Michel, V.; Thirion, B.; Grisel, O.; Blondel, M.; Prettenhofer, P.; Weiss, R.; Dubourg, V.; Vanderplas, J.; Passos, A.; Cournapeau, D.; Brucher, M.; Perrot, M.; Duchesnay, E. Scikit-learn: Machine Learning in Python. *J Mach Learn Res* **2011**, *12*, 2825–2830.

(S20) Moulavi, D.; Jaskowiak, P. A.; Campello, R. J. G. B.; Zimek, A.; Sander, J. Density-Based Clustering Validation. Proceedings of the 2014 SIAM International Conference on Data Mining. 2014.

(S21) Humphrey, W.; Dalke, A.; Schulten, K. VMD – Visual Molecular Dynamics. *J. Mol. Graph.* **1996**, *14*, 33–38.

(S22) Cupellini, L.; Calvani, D.; Jacquemin, D.; Mennucci, B. Charge transfer from the carotenoid can quench chlorophyll excitation in antenna complexes of plants. *Nat. Commun.* **2020**, *11*, 1–8.

(S23) Cignoni, E.; Lapillo, M.; Cupellini, L.; Acosta-Gutiérrez, S.; Gervasio, F. L.; Mennucci, B. A different perspective for nonphotochemical quenching in plant antenna complexes. *Nat. Commun.* **2021**, *12*, 1–9.

(S24) Lapillo, M.; Cignoni, E.; Cupellini, L.; Mennucci, B. The energy transfer model of non-photochemical quenching: Lessons from the minor CP29 antenna complex of plants. *Biochim. Biophys. Acta (BBA) - Bioenerg.* **2020**, *1861*, 148282.
